# Supplementary material for: Amplification of integrated microscopic motions of high-density [2]rotaxanes in mechanically interlocked networks
Source: Nat Commun. 2022 Nov 4;13:6654. doi: 10.1038/s41467-022-34286-6 (PMC9636211; doi:10.1038/s41467-022-34286-6)
Supplement: Supplementary file 1 — Supplementary Information [file 41467_2022_34286_MOESM1_ESM.pdf]

Supplementary information for

Amplification of integrated mechanical motions of high-density [2]rotaxane moieties in mechanically interlocked networks

Xue Yang[1†], Lin Cheng[1†], Zhaoming Zhang[1†], Jun Zhao[1], Ruixue Bai[1], Zhewen Guo[1], Wei Yu[1*], and Xuzhou Yan[1*]

[1]School of Chemistry and Chemical Engineering, Frontiers Science Center for Transformative Molecules, Shanghai Jiao Tong University, Shanghai 200240, P. R. China

[†]These authors contributed equally to this work.

[*]Corresponding authors. E-mails: wyu@sjtu.edu.cn; xzyan@sjtu.edu.cn

Supplementary Methods

All reagents were commercially available and used as supplied without further purification. All the solvents were used as received from Adamas®beta with a Reagent Grade. Deuterated solvents were purchased from Cambridge Isotope Laboratory (Andover, MA). All reactions were performed at ambient laboratory conditions, and no precautions are taken to exclude atmospheric moisture unless otherwise specified. Compounds **5**[S1], **10**[S2], **12**[S3], and **15**[S4] were prepared according to the literature procedures. For these reported compounds, only ^{1}H NMR spectra were measured and compared with those in literatures to confirm their structures.

Nuclear magnetic resonance (NMR) spectra were recorded with a Bruker Avance DMX 400 spectrophotometer with use of the deuterated solvent as the lock and the residual solvent or TMS as the internal reference. ^{1}H and ^{13}C NMR chemical shifts were reported relative to residual solvent signals. High resolution mass spectra were obtained on a Bruker SolariX 7.0T FT-ICR MS spectrometer.

Fourier transform infrared (FTIR) spectroscopy measurements were performed on a Thermoscientific Nicolet 6700 FTIR spectrometer at room temperature. The spectra were collected through 32 scans with a spectral resolution of 4 cm^{-1} over a wavenumber range of 4000~600 cm^{-1}.

The thermal stability analysis was conducted using a TA Instruments Q500 thermogravimetric analyzer (TGA) under the nitrogen. Each sample (5~10 mg) was heated from 50 to 700 °C with a rate of 10 °C/min. Transition temperatures of materials determined on a TA Instruments 2500 differential scanning calorimetry (DSC) under the nitrogen. Each sample (5~10 mg) was heated from −50 to 150 °C with a rate of 10 °C/min.

Dynamic mechanical analyses (DMA) were performed on the Discovery DMA Q850 apparatus (Germany) with a tensile mode. Temperature ramp measurements (−50~100 °C) at a heating rate of 5.0 °C/min were carried out with an oscillation frequency of 1.0 Hz, an oscillation amplitude of 10 μm, and an automatic tension setting of 125%.

Tensile tests

The tensile stress−strain measurements were performed using a tensile tester (Instron 3343 with a 100 N sensor) in air at room temperature. Unless otherwise noted, the tensile curves were

measured at a constant speed of 100 mm/min. Young's modulus was determined from the initial slope of the stress–strain curves. The toughness, a parameter that characterizes the work required to fracture the sample per unit, was calculated from the area below the tensile stress–strain curve until fracture. Energy dissipation was calculated by integrating the area encompassed by the cyclic tensile curves. Damping capacity was defined as the ratio of the dissipated energy (the area encompassed by the loading and unloading curves) to the loading energy (the area encompassed by the loading curve). Cyclic tensile tests for the recovery experiments were performed with a predefined strain of 100% or 200% at a deformation rate of 100 mm/min.

Swelling tests

The swelling experiments were performed with a piece of sample (~100 mg). The samples were immersed in hexane, chloroform ($CHCl_3$), dichloromethane (CH_2Cl_2), tetrahydrofuran (THF), acetone, acetonitrile, and dimethylformamide (DMF) at room temperature, respectively. The swelling ratio was calculated by Equation 1:

$$\text{Swelling ratio (\%)} = \left[(W_\mathrm{s} - W_\mathrm{d})/W_\mathrm{d}\right] \times 100 \qquad (1)$$

where W_s is the weight of the swollen sample, W_d is the weight of the dry sample.

Puncture resistance tests

The samples were fixed between two plates with a distance of 5.0 mm to let the samples deform freely during the test. The as-prepared samples were cut into 5 mm (width) × 15 mm (length) × 0.3 mm (thickness) rectangles for the puncture resistance measurements. A toothpick was positioned perpendicularly and moved down to the sample in a tensile test instrument with a speed at 50 mm/min. The tip diameter of the toothpick was 400 μm, which was measured by polarizing optical microscope (Leica DM LP). The puncture resistance was evaluated by maximal puncture force, displacement, and energy. The puncture energy (E) was calculated by the force-displacement curve integral from the Equation 2:

$$E = \int F \, dl \qquad (2)$$

where F is the puncture force at a displacement l.

Rheological tests

Rheological experiments were carried out using a TA Instruments ARES G2 stress-controlled rheometer with an 8.0 mm parallel plate attachment. The as-prepared samples were cut into circles with a thickness of 0.5 mm and diameter of 8 mm for the rheological measurements. Cyclic temperature sweeps were performed from 30 to 80 °C with a heating/cooling rate of 5 °C/min under the frequency of 1.0 rad/s. The continuous multi-step stress relaxation (SR) tests were measured at 80 °C, and each stage (SR-1−5) was tested for 100 s. At SR-2 stage, the defined constant strain values (γ_0) are 1, 3, 5, 7, 9, 11, 13, 15, 17, 19, 20, 21, 23, 25, 27, 29, 30, 31, 33, 35, 37, 40, 45, 50, 55, 60, 70, 80, 90, 100, 150, 300%, respectively. The combined rheological tests were implemented at 80 °C. The shear strain (γ_0) and shear rate ($\dot{\gamma}$) can be changed by adjusting the time (t), as shown in Table S1.

Supplementary Table 1 The time (t), shear strain (γ_0), and shear rate ($\dot{\gamma}$) of the combined rheological tests.

t (s)	γ_0 (%)	$\dot{\gamma}$ (s^{-1})
1.0	1.0	0.020
3.2	10	0.063
4.0	16	0.080
7.0	49	0.14
10	100	0.20
16	256	0.32
20	400	0.40
22	484	0.44
27	729	0.54
32	1024	0.64

Constrained geometries simulate external force calculations

The constrained geometries simulate external force (CoGEF) calculations were performed on Spartan'14 following Beyer's method [S5]. To clarify the force induced dynamicity process, the two terminal alkyl chains on the designed molecule were simplified as methyl groups. The initial structure was built in Spartan'14 and minimized using molecular mechanics (MMFF). In the CoGEF process, the distance between the methyl groups was constrained and increased by increments of 0.1 Å. At each step, the energy was minimized by molecular mechanics (MMFF) via Spartan'14 in vacuum without counterion, and then DFT via Gaussian09 (G09)[S6] with the Becke three-parameter hybrid exchange and the Lee−Yang−Parr correlation functional (B3LYP)[S7,S8] and the 6-31G* basis set[S9]. The relative energy of each intermediate was determined by setting the energy of the initial state at 0 kJ/mol.

Procedure for the preparation of MINs-1−7 and control

Typical procedure for the MIN-**4** (molar ratio of DODT/PETMP = 3/1): In a vial, [2]rotaxane **1** (300 mg, 0.25 mmol), 3,6-dioxa-1,8-octandithiol (DODT) (27.0 mg, 0.15 mmol), and pentaerythritol tetrakis(3-mercaptopropionate) (PETMP) (24.0 mg, 0.05 mmol) were dissolved in 1.0 mL of CH_2Cl_2, and then the photo initiator 2,2-dimethoxy-2-phenylacetophenone (DMPA) (1.0 wt%) was added to the solution. The mixture was stirred at room temperature for 10 min. Then the solution was poured into a mold and irradiated under UV light (365 nm, 20 mW/cm^2) for 30 min. Afterwards, the MIN-**4** was put in an oven at 60 °C for 12 h to further cure the sample. Others six MINs (molar ratios of DODT/PETMP = 0/1, 0.3/1, 1/1, 8/1, 15/1, and 1/0, respectively) were prepared according to the same procedure. Besides, the control containing the same thiol feeding ratio with MIN-**4** was prepared according to the same procedure for comparison.

Synthetic route of [2]rotaxane 1

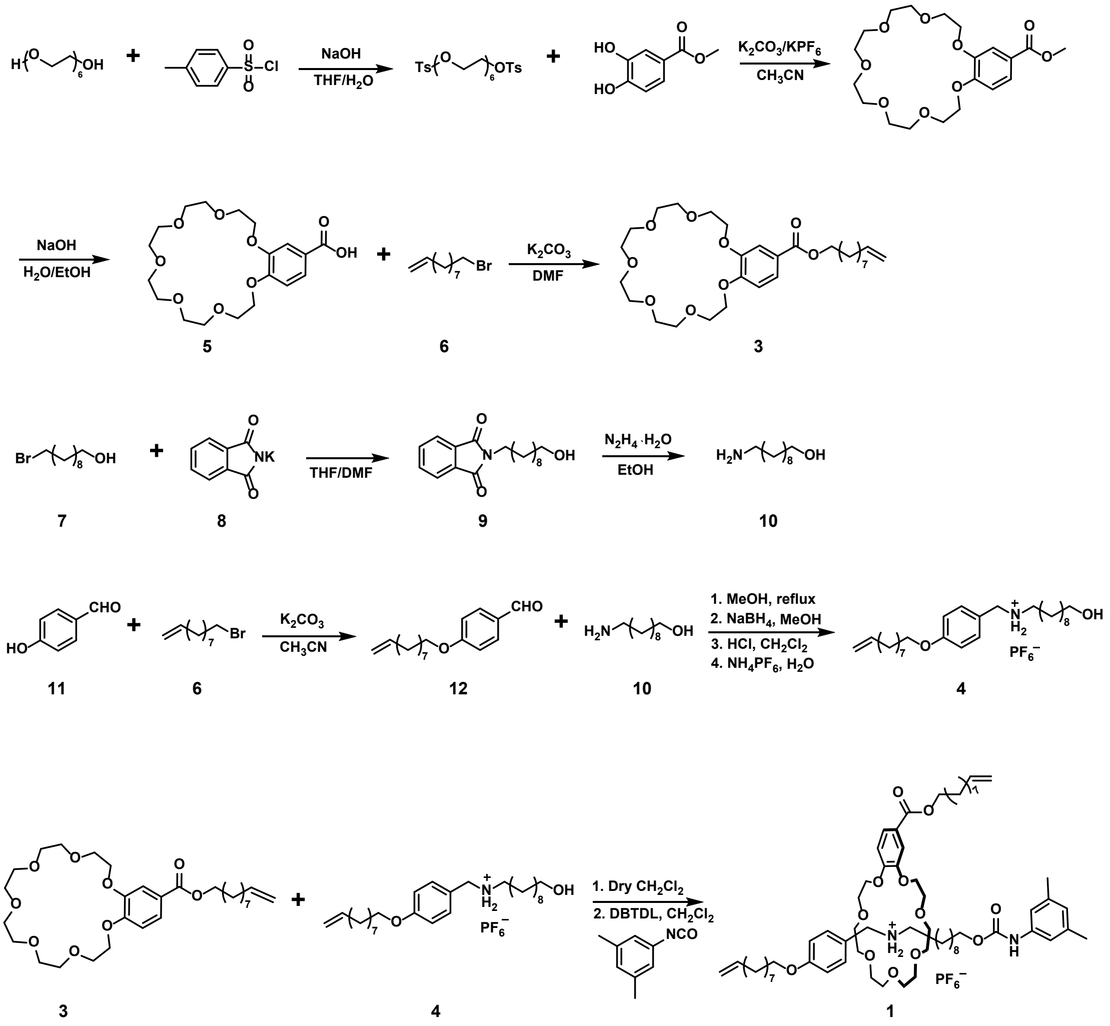

Supplementary Fig. 1 Synthetic route of [2]rotaxane **1**.

Synthesis of compound 3

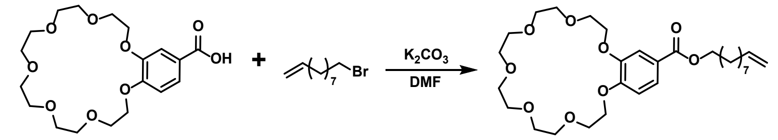

To a solution of **5** (10.0 g, 25.0 mmol) and 10-bromo-1-decene **6** (5.47 g, 25.0 mmol) in DMF (300 mL), K_2CO_3 (5.20 g, 37.5 mmol) was added at room temperature. The stirred mixture was heated at 80 °C for 12 h. The reaction mixture was cooled to room temperature and filtered, and concentrated to give a yellow oil, which was then purified by flash column chromatography (ethyl acetate:petroleum ether, 9:1 *v*/*v*) to afford compound **3** as a yellow oil (10.7 g, 80%). The ^{1}H NMR spectrum of compound **3** is shown in Figure S1. ^{1}H NMR (400 MHz, $CDCl_3$, 298K) δ (ppm): 7.65 (d, J = 8.4 Hz, 1H), 7.54 (s, 1H), 6.86 (dd, J = 8.5, 1.2 Hz, 1H), 5.87–5.73 (m, 1H), 5.03–4.89 (m, 2H), 4.27 (t, J = 6.8 Hz, 2H), 4.23–4.16 (m, 4H), 4.22–4.19 (m, 4H), 3.82–3.77 (m, 4H), 3.76–3.70 (m, 4H), 3.66–3.69 (m, 8H), 2.08–1.99 (m, 2H), 1.77–1.68 (m, 2H), 1.45–1.20 (m, 10H). The ^{13}C NMR spectrum of compound **3** is shown in Figure S2. ^{13}C NMR (100 MHz, $CDCl_3$, 298K) δ (ppm): 166.35, 152.78, 148.20, 139.09, 123.80, 123.28, 114.59, 114.15, 112.24, 71.24, 71.13, 71.05, 71.02, 70.95, 70.53, 69.61, 69.46, 69.22, 69.02, 64.92, 33.73, 29.32, 29.19, 29.00, 28.97, 28.90, 28.84, 28.72, 25.99. HR-ESI-MS is shown in Figure S3: *m*/*z* calcd for $C_{29}H_{46}O_9$, 556.3480 $[M + NH_4]^+$; found 556.3491 $[M + NH_4]^+$.

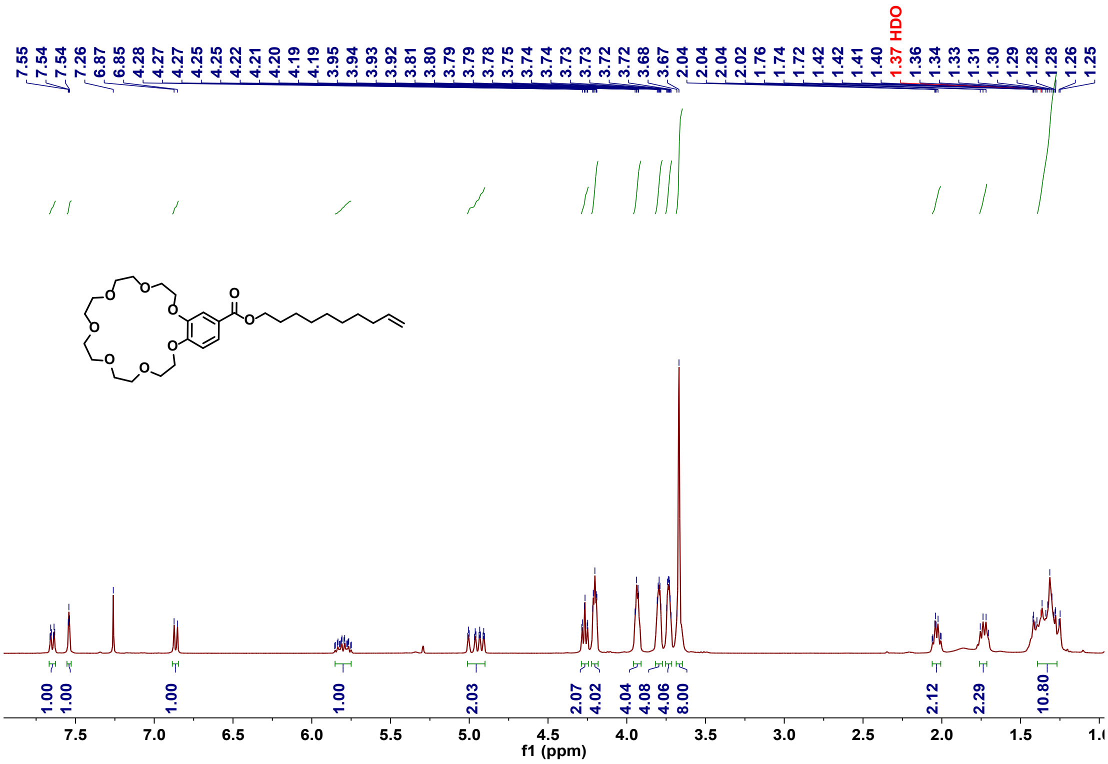

Supplementary Fig. 2 ^{1}H NMR spectrum (400 MHz, $CDCl_3$, 298K) of compound **3**.

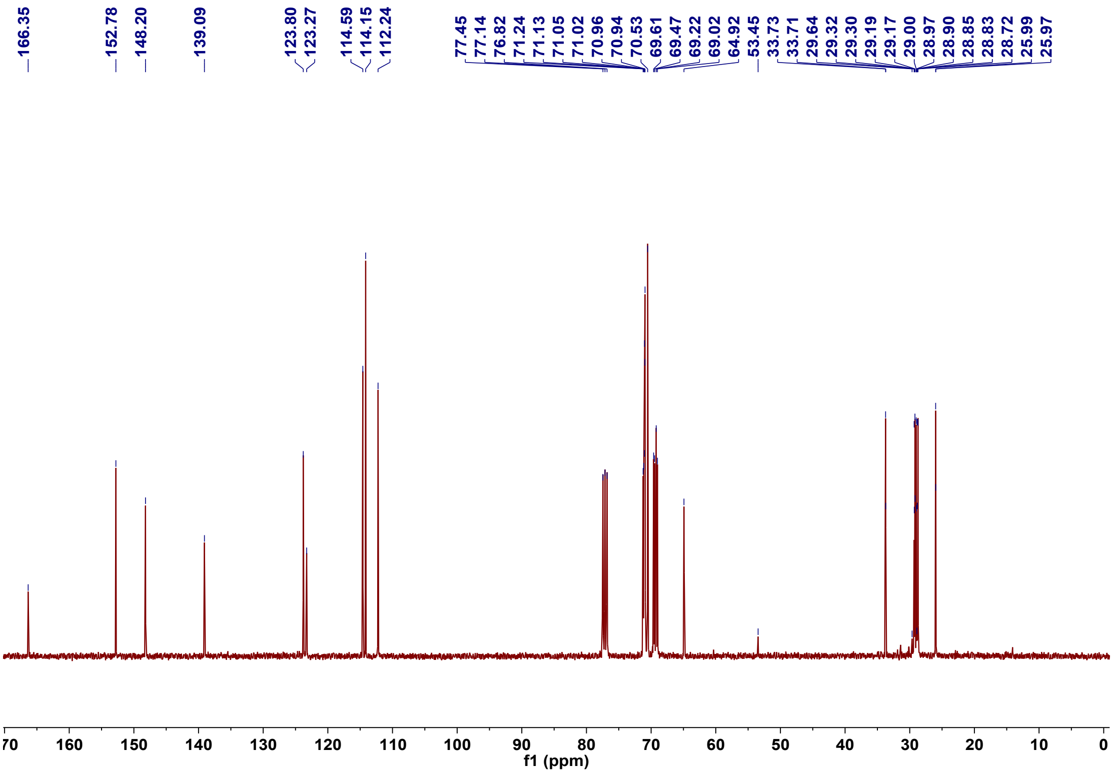

Supplementary Fig. 3 ^{13}C NMR spectrum (100 MHz, $CDCl_3$, 298K) of compound **3**.

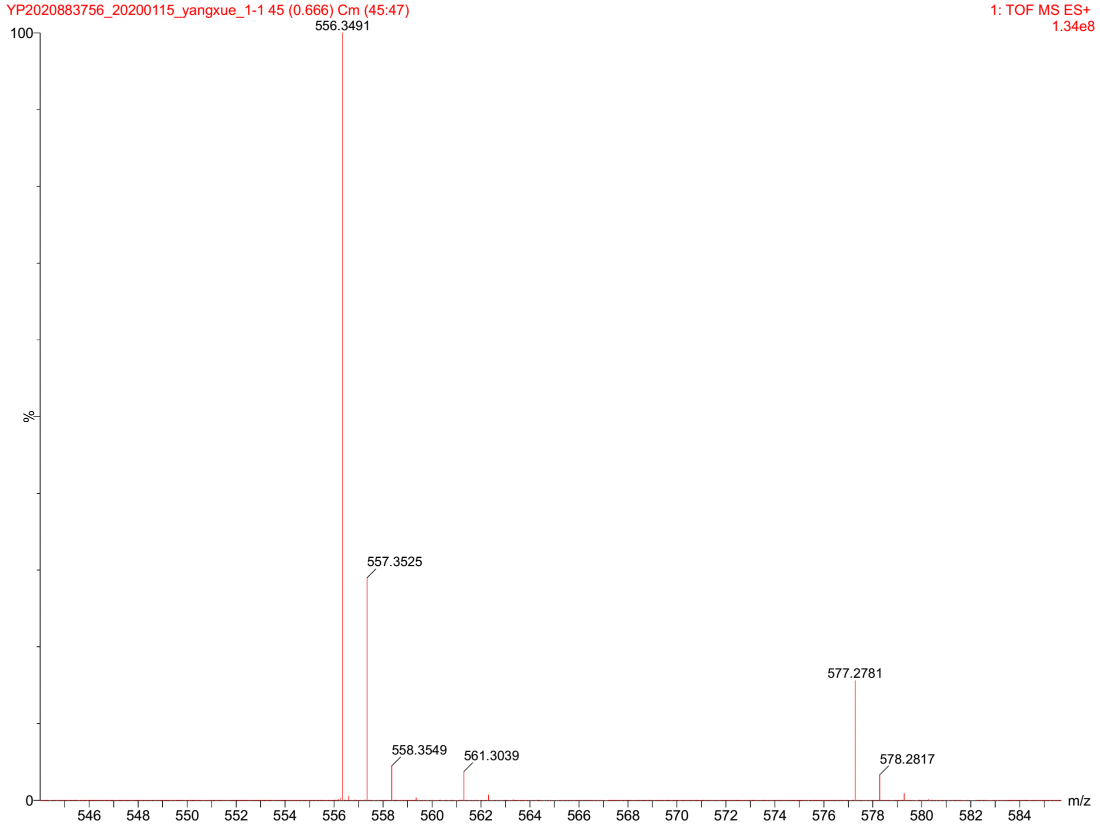

Supplementary Fig. 4 Electrospray ionization mass spectrum of compound **3**.

Synthesis of compound 12 (ref. S3)

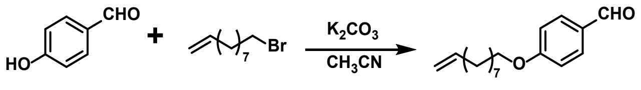

11 6 12

To a solution of 4-hydroxy benzaldehyde **11** (6.10 g, 50.0 mmol) and 10-bromo-1-decene **6** (11.5 g, 52.5 mmol) in CH_3CN (300 mL), K_2CO_3 (7.60 g, 55.0 mmol) was added at room temperature. The stirred mixture was heated at 65 °C for 12 h. The reaction mixture was cooled to room temperature, filtered, and concentrated to give a pale yellow oil, which was then purified by flash column chromatography (ethyl acetate:petroleum ether, 1:7 *v*/*v*) to afford compound **12** as a pale yellow oil (12.7 g, 98%). The ^{1}H NMR spectrum of compound **12** is shown in Figure S4. ^{1}H NMR (400 MHz, $CDCl_3$, 298K) δ (ppm): 9.87 (s, 1H), 7.82 (d, J = 8.8 Hz, 2H), 6.98 (d, J = 8.8 Hz, 2H), 5.87–5.74 (m, 1H), 5.03–4.90 (m, 2H), 4.03 (t, J = 6.8 Hz, 2H), 2.08–2.00 (m, 2H), 1.85–1.76 (m, 2H), 1.50–1.26 (m, 10H).

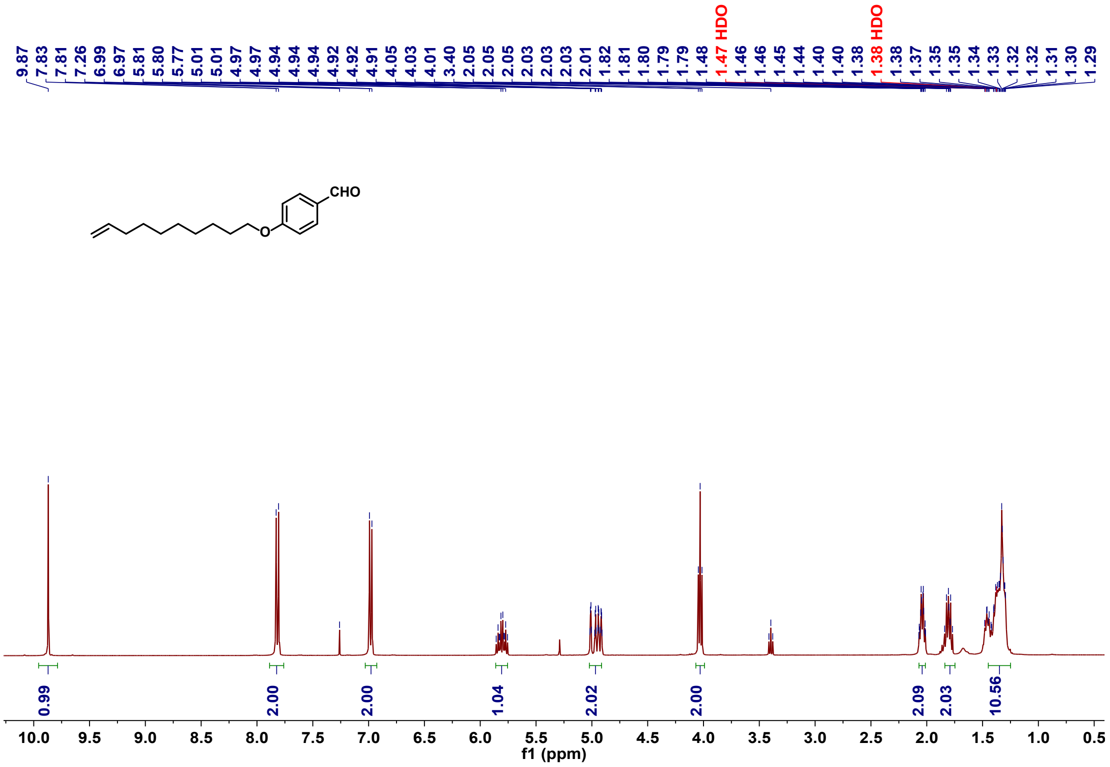

Supplementary Fig. 5 ^{1}H NMR spectrum (400 MHz, $CDCl_3$, 298K) of compound **12**.

Synthesis of compound 4

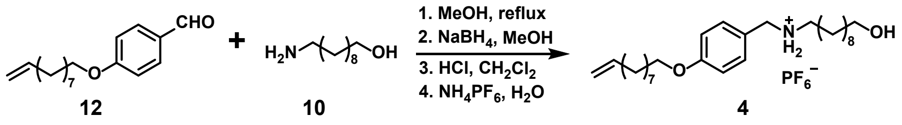

A solution of **12** (3.00 g, 11.5 mmol) and **10** (2.09 g, 12.0 mmol) was refluxed overnight in MeOH (200 mL). After the reaction mixture was cooled to ambient temperature, $NaBH_4$ (0.87 g, 23.0 mmol) was added portionwise to the stirring solution over a period of 0.5 h. Stirring was maintained under ambient conditions for further 24 h, after which time H_2O was added to neutralize excess $NaBH_4$. The mixture was filtered and MeOH was removed with a rotary evaporator. The residue was extracted with ethyl acetate and the organic phase was concentrated to yield a yellow oil. After the oil was added to a hydrochloric acid solution and stirred for a moment, a white precipitate formed. The mixture was filtered and the solid was dissolved in hot water. The solution was added to a saturated aqueous NH_4PF_6 solution to produce a precipitate, which was collected by suction filtration to afford compound **4** (3.38 g, 52%) as a white solid. M.p. = 68 °C. The ^1H NMR spectrum of compound **4** is shown in Figure S5. ^1H NMR (400 MHz, $CDCl_3$, 298K) δ (ppm): 7.32 (d, J = 8.4 Hz, 2H), 6.90 (d, J = 8.8 Hz, 2H), 5.86–5.75 (m, 1H), 5.02–4.91 (m, 2H), 4.09 (s, 2H), 3.91 (t, J = 6.4 Hz, 2H), 3.59 (t, J = 6.8 Hz, 2H), 2.87 (t, J = 7.6 Hz, 2H), 2.08–2.00 (m, 2H), 1.80–1.71 (m, 2H), 1.70–1.61 (m, 2H), 1.58–1.47 (m, 2H), 1.46–1.21 (m, 22H). The ^{13}C NMR spectrum of compound **4** is shown in Figure S6. ^{13}C NMR (100 MHz, $CDCl_3$, 298K) δ (ppm): 160.34, 139.15, 131.60, 121.19, 115.19, 114.19, 68.10, 62.94, 51.44, 47.04, 33.78, 32.40, 29.67, 29.41, 29.35, 29.17, 29.06, 29.05, 28.90, 28.83, 28.68, 26.08, 26.00, 25.79, 25.45. HRESIMS is shown in Figure S7: m/z calcd for $C_{27}H_{48}NO_2$, 418.3680 $[M - PF_6]^+$; found 418.36641 $[M - PF_6]^+$.

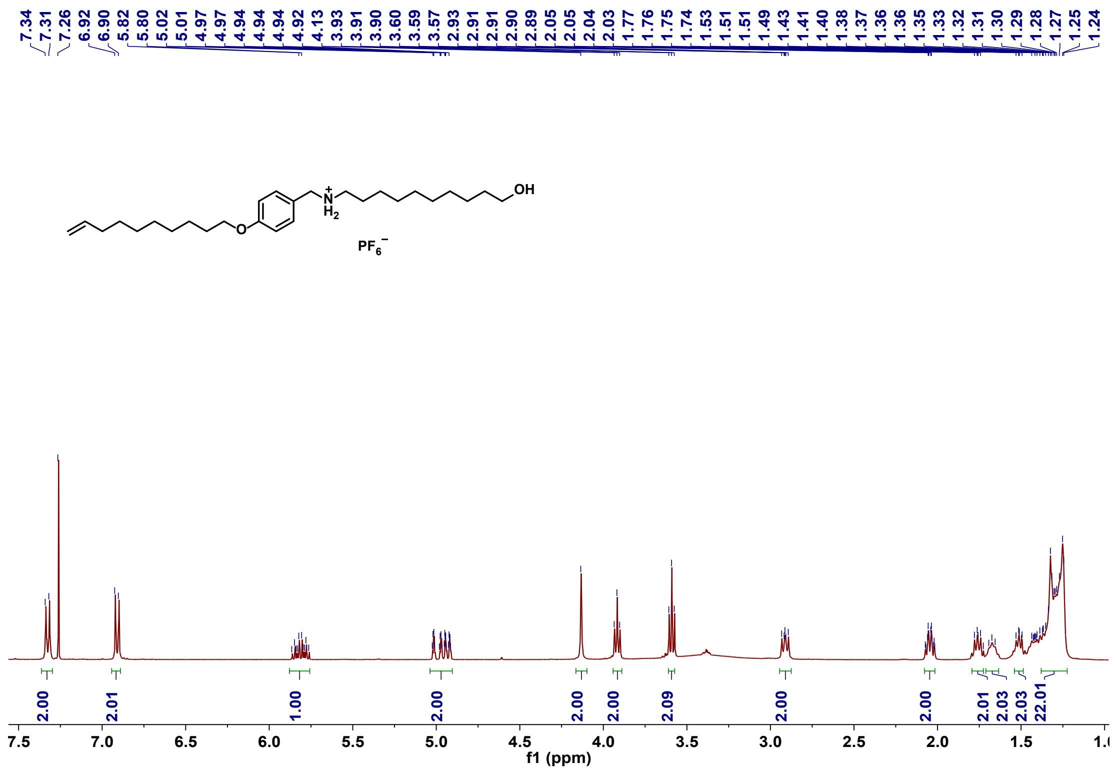

Supplementary Fig. 6 ^{1}H NMR spectrum (400 MHz, $CDCl_3$, 298K) of compound **4**.

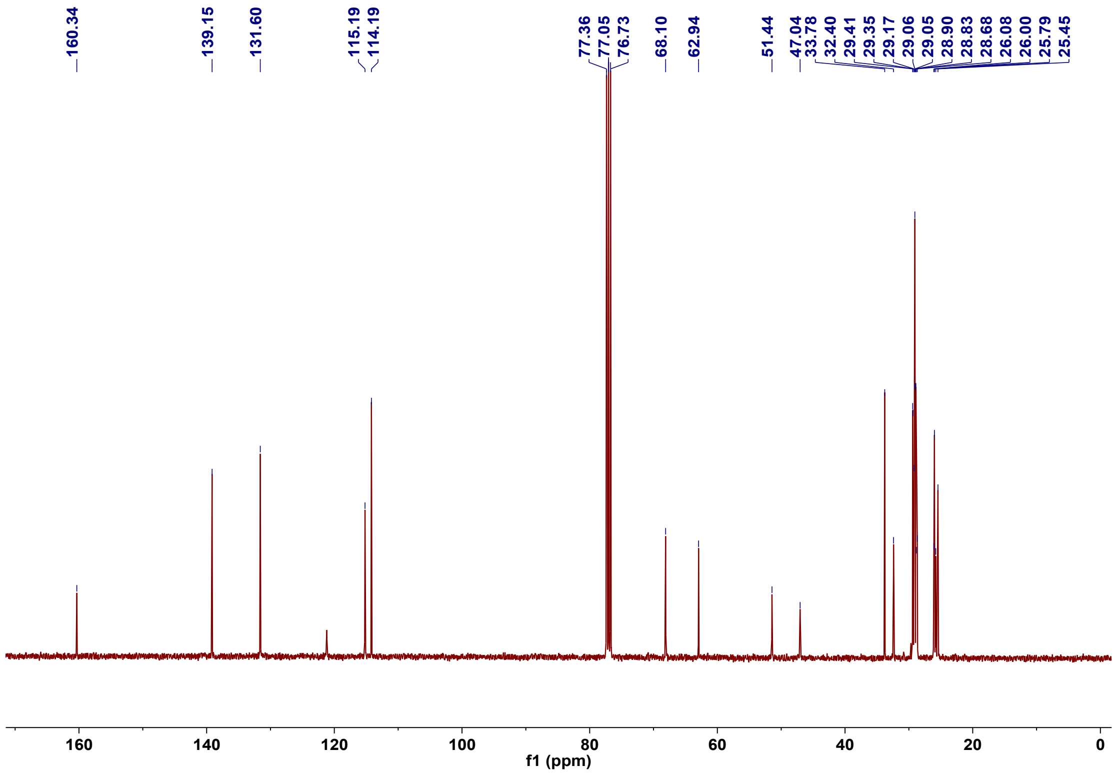

Supplementary Fig. 7 ^{13}C NMR spectrum (100 MHz, $CDCl_3$, 298K) of compound **4**.

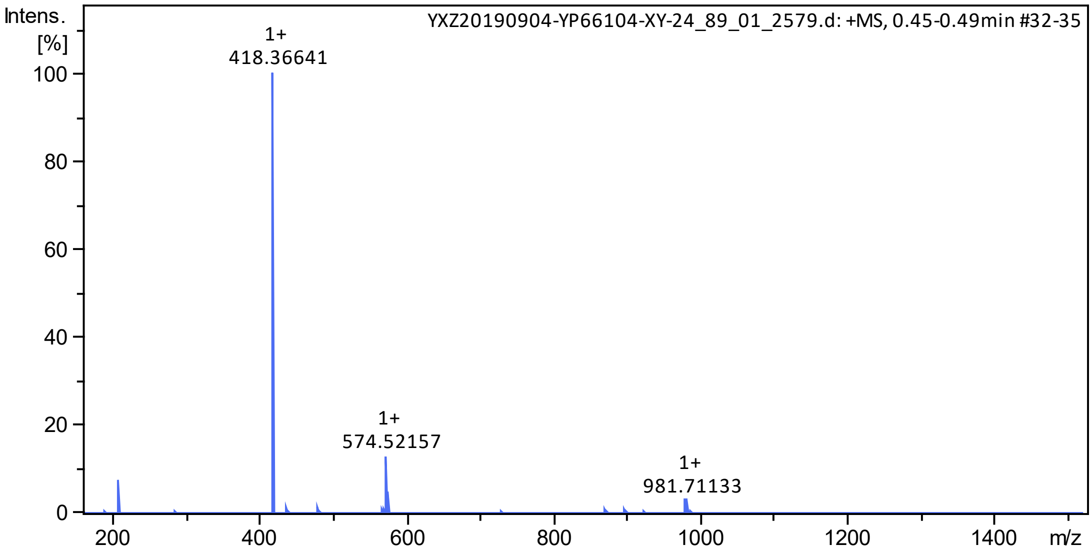

Supplementary Fig. 8 Electrospray ionization mass spectrum of compound **4**.

Synthesis of [2]rotaxane 1

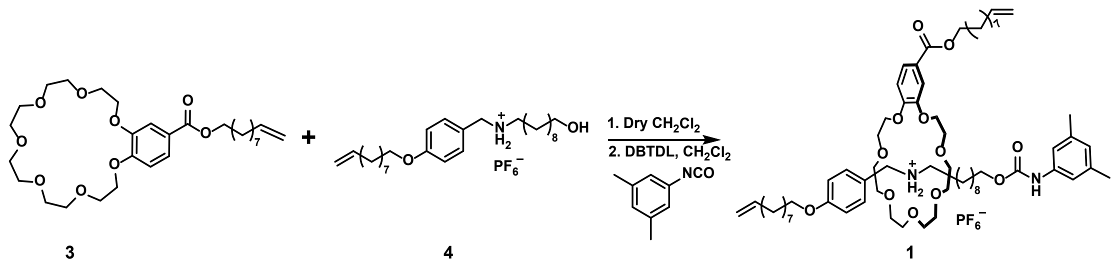

A solution of **3** (2.87 g, 5.33 mmol) and **4** (2.82 g, 5.00 mmol) in dry CH_2Cl_2 (250 mL) was heated under reflux overnight. Upon cooling to room temperature, catalytic amount of dibutyltin dilaurate (DBTDL, 19.0 mg) and 3,5-dimethylphenyl isocyanate (1.47 g, 10.0 mmol) were added to the solution which was stirred at room temperature for two days. Upon removal of CH_2Cl_2, a yellow oil was obtained, which was purified by flash column chromatography (MeOH:CH_2Cl_2, 1:100 *v*/*v*) to afford compound **1** as a yellow oil (3.48 g, 57%). The ^{1}H NMR spectrum of compound **1** is shown in Figure S8. ^{1}H NMR (400 MHz, $CDCl_3$, 298K) δ (ppm): 7.69 (dd, J = 8.4, 2.0 Hz, 1H), 7.54 (s, 1H), 7.47 (s, 2H), 7.18 (d, J = 8.8 Hz, 2H), 7.03 (s, 2H), 6.95 (d, J = 8.4 Hz, 1H), 6.85 (d, J = 8.8 Hz, 2H), 6.69 (s, 1H), 5.86–5.73 (m, 2H), 5.02–4.88 (m, 4H), 4.39–4.31 (m, 2H), 4.31–4.21 (m, 6H), 4.15–4.08 (m, 2H), 3.99–3.80 (m, 6H), 3.75–3.36 (m, 18H), 2.27 (s, 6H), 2.06–2.00 (m, 4H), 1.79–1.71 (m, 4H), 1.67–1.60 (m, 4H), 1.55–1.21 (m, 32H). The ^{13}C NMR spectrum of compound **1** is shown in Figure S9. ^{13}C NMR (100 MHz, $CDCl_3$, 298K) δ (ppm): 166.18, 159.88, 153.85, 150.82, 146.62, 139.19, 139.15, 138.69, 137.96, 131.34, 124.98, 124.14, 123.68, 116.36, 114.70, 114.18, 114.16, 112.47, 111.46, 71.56, 71.12, 70.79, 70.61, 70.52, 69.63, 69.55, 69.03, 68.50, 68.18, 65.25, 65.11, 50.47, 46.98, 33.78, 29.40, 29.38, 29.36, 29.32, 29.23, 29.18, 29.16, 29.06, 29.04, 28.90, 28.88, 28.73, 27.04, 26.66, 25.98, 25.79, 21.39. HR-ESI-MS is shown in Figure S10: *m*/*z* calcd for $C_{65}H_{103}N_2O_{12}$, 1103.7506 $[M - PF_6]^+$; found 1103.75338 $[M - PF_6]^+$.

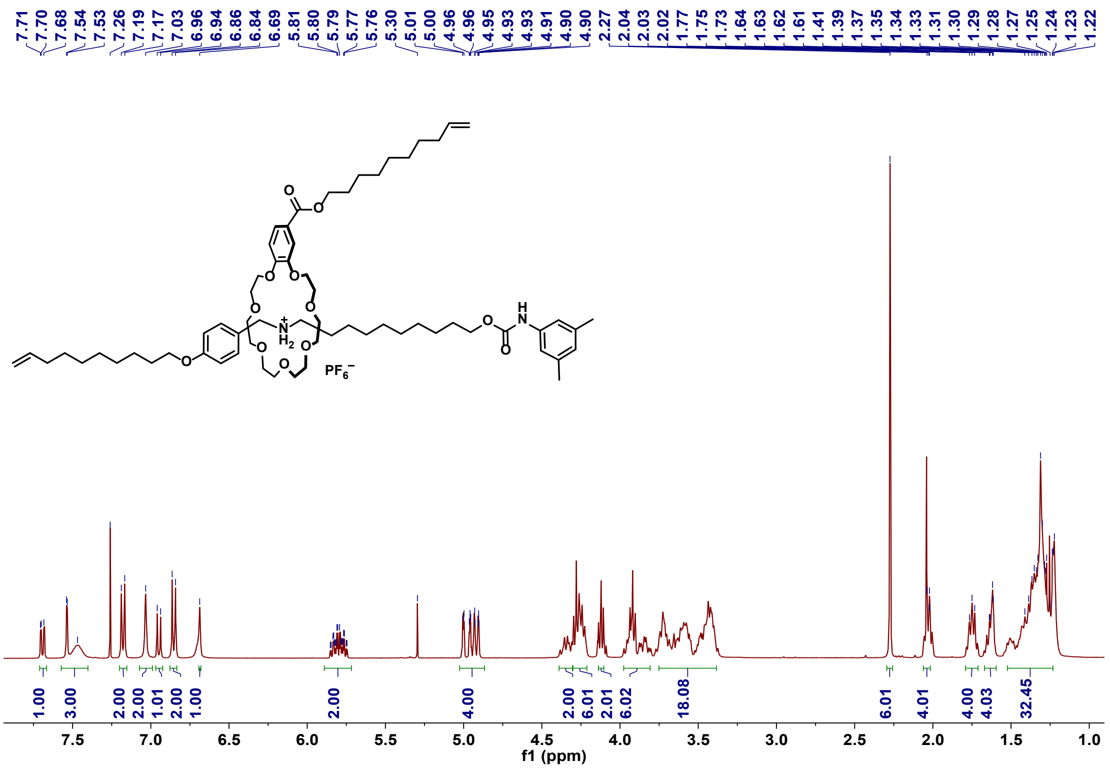

Supplementary Fig. 9 ^{1}H NMR spectrum (400 MHz, $CDCl_3$, 298K) of compound **1**.

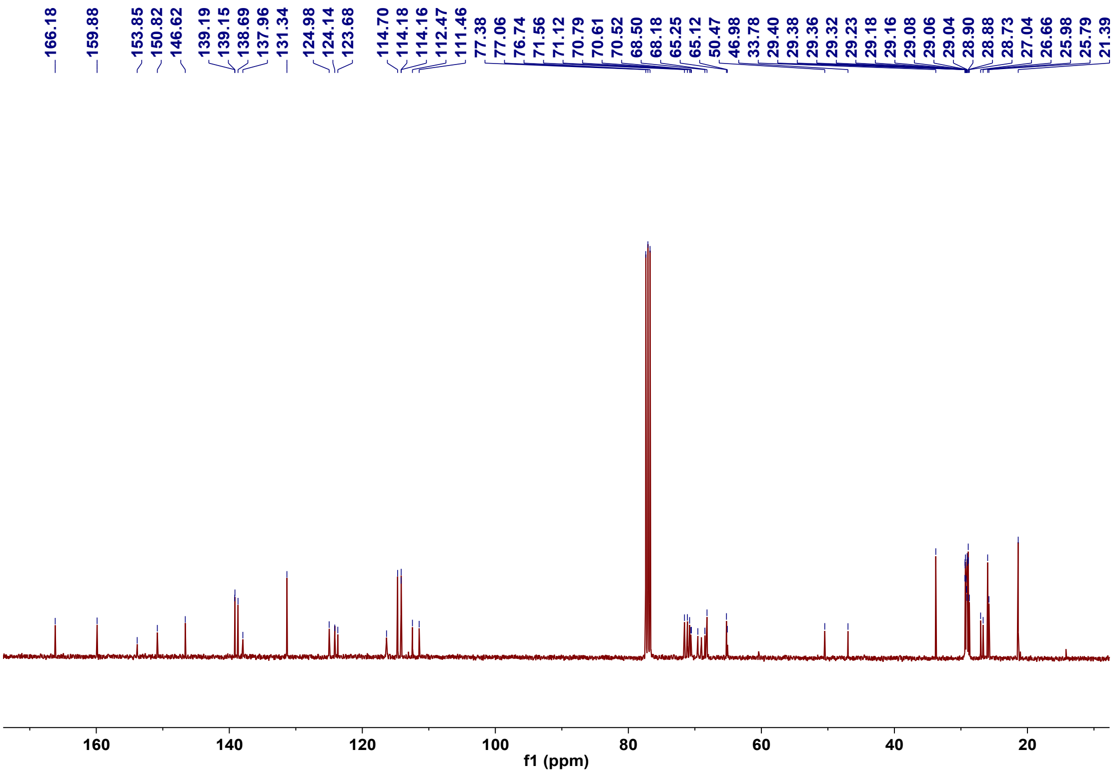

Supplementary Fig. 10 ^{13}C NMR spectrum (100 MHz, $CDCl_3$, 298K) of compound **1**.

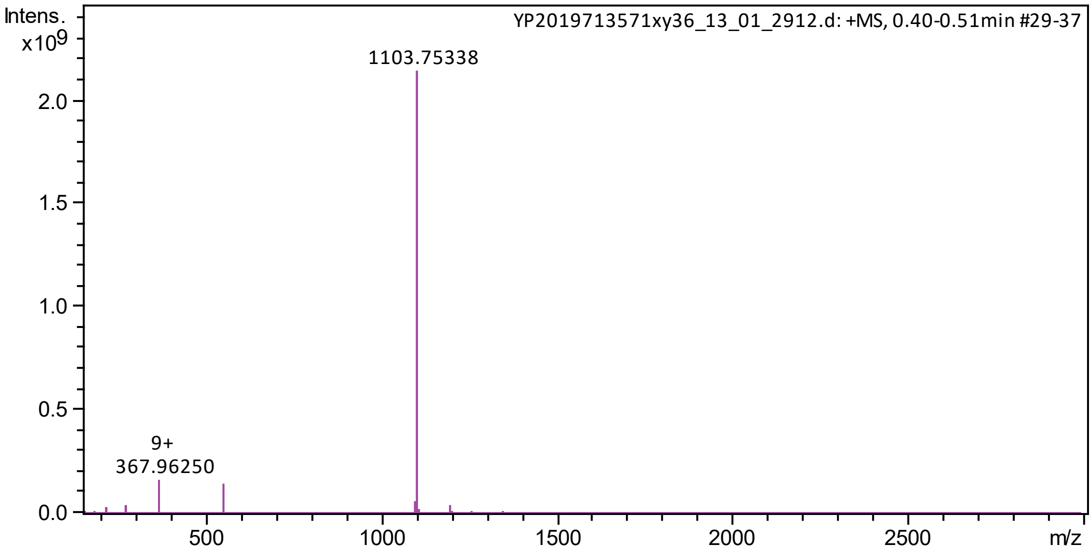

Supplementary Fig. 11 Electrospray ionization mass spectrum of compound **1**.

Synthetic route of [2]rotaxane 2

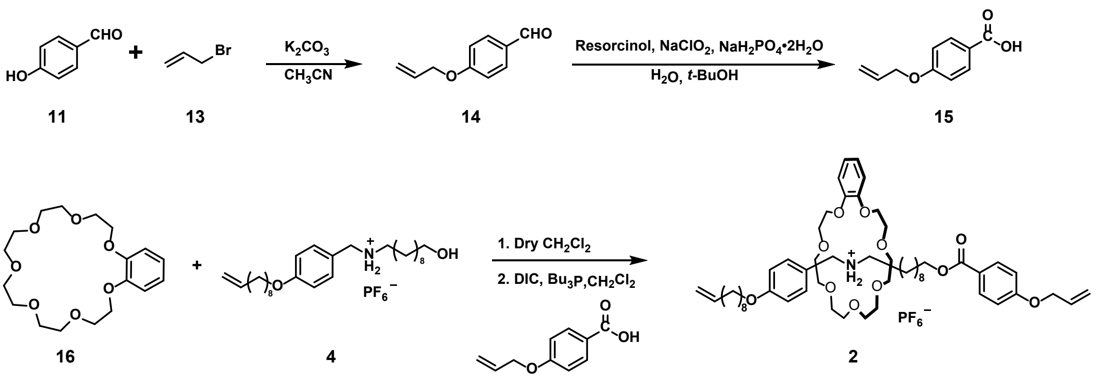

Supplementary Fig. 12 Synthetic route of [2]rotaxane **2**.

Synthesis of [2]rotaxane 2

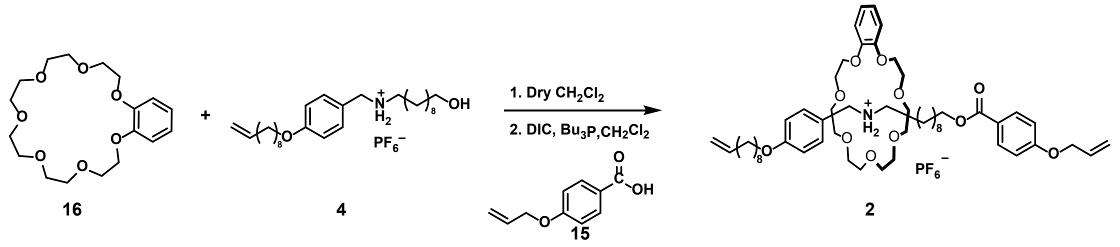

A solution of **16** (1.00 g, 2.81 mmol) and **4** (1.58 g, 2.81 mmol) in dry CH_2Cl_2 (100 mL) was heated under reflux overnight. Upon cooling to room temperature, *N,N'*-diisopropylcarbodiimide (DIC, 1.06 g, 8.43 mmol), tributyl phosphine (Bu_3P, 0.57 g, 2.81 mmol), and **15** (1.00 g, 5.62 mmol) were added to the solution, which was then stirred at room temperature for 24 h. The reaction mixture was poured into hexane, and the precipitates were collected by decantation and purified by column chromatography (MeOH:CH_2Cl_2, 1:100 *v*/*v*) to afford compound **2** as a yellow oil (1.21 g, 40%). The ^1H NMR spectrum of compound **2** is shown in Figure S11. ^1H NMR (400 MHz, $CDCl_3$, 298K) δ (ppm): 7.98 (d, J = 8.8 Hz, 2H), 7.44 (s, 2H), 7.19 (d, J = 8.4 Hz, 2H), 6.96–6.81 (m, 8H), 6.10–5.98 (m, 1H), 5.86–5.74 (m, 1H), 5.45–5.30 (m, 2H), 5.02–4.90 (m, 2H), 4.59 (d, J = 5.6 Hz, 2H), 4.31–4.15 (m, 8H), 3.95–3.87 (m, 4H), 3.82–3.54 (m, 12H), 3.53–3.40 (m, 8H), 2.07–1.98 (m, 2H), 1.81–1.18 (m, 28H). The ^{13}C NMR spectrum of compound **2** is shown in Figure S12. ^{13}C NMR (100 MHz, $CDCl_3$, 298K) δ (ppm): 166.46, 166.32, 159.84, 146.78, 139.19, 132.58, 131.51, 131.38, 124.33, 123.01, 121.60, 118.11, 114.66, 114.33, 114.15, 111.76, 71.45, 71.11, 70.92, 70.60, 69.75, 68.88, 68.18, 64.74, 53.45, 50.47, 46.96, 33.78, 29.39, 29.37, 29.20, 29.18, 29.11, 29.06, 28.90, 28.75, 27.06, 25.99. HR-ESI-MS is shown in Figure S13: *m/z* calcd for $C_{55}H_{84}NO_{11}$, 934.6039 $[M - PF_6]^+$; found 934.6089 $[M - PF_6]^+$.

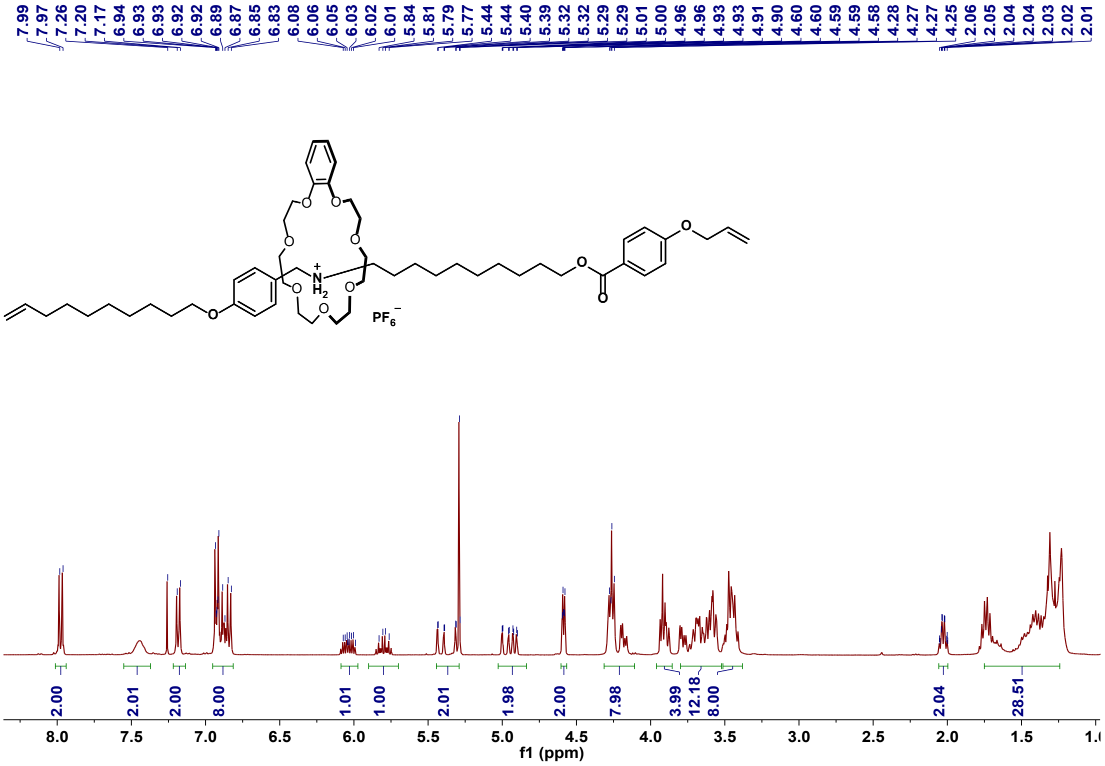

Supplementary Fig. 13 ^{1}H NMR spectrum (400 MHz, $CDCl_3$, 298K) of compound **2**.

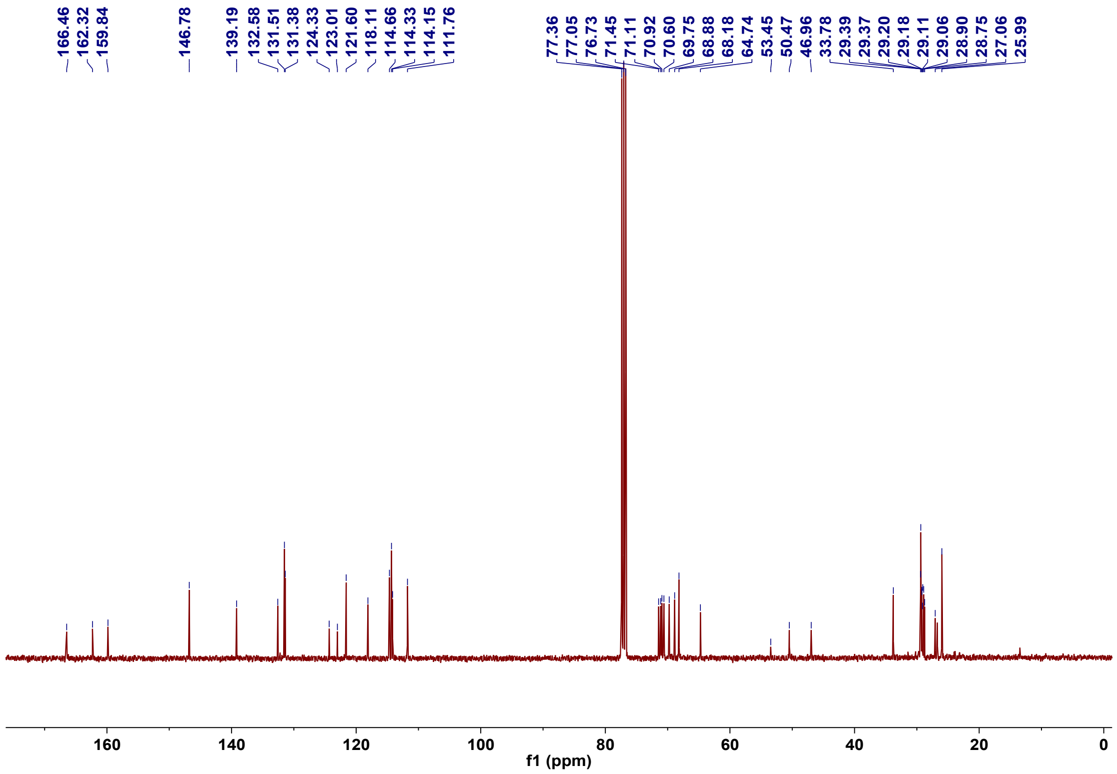

Supplementary Fig. 14 ^{13}C NMR spectrum (100 MHz, $CDCl_3$, 298K) of compound **2.**

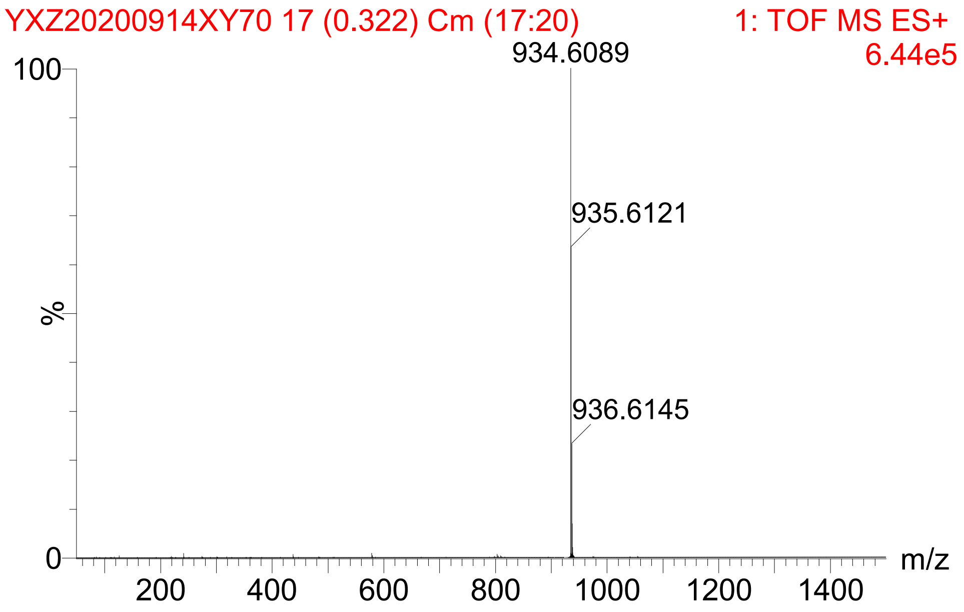

Supplementary Fig. 15 Electrospray ionization mass spectrum of compound **2**.

Supplementary Discussion

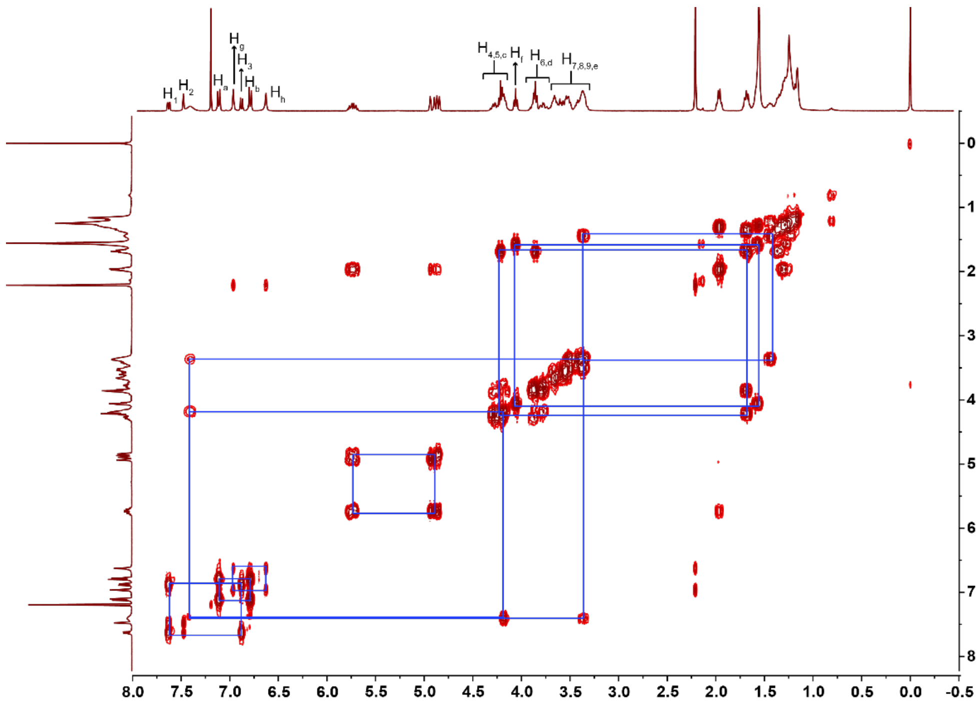

Supplementary Fig. 16 COSY NMR spectrum (400 MHz, $CDCl_3$, 298K) of [2]rotaxane **1**.

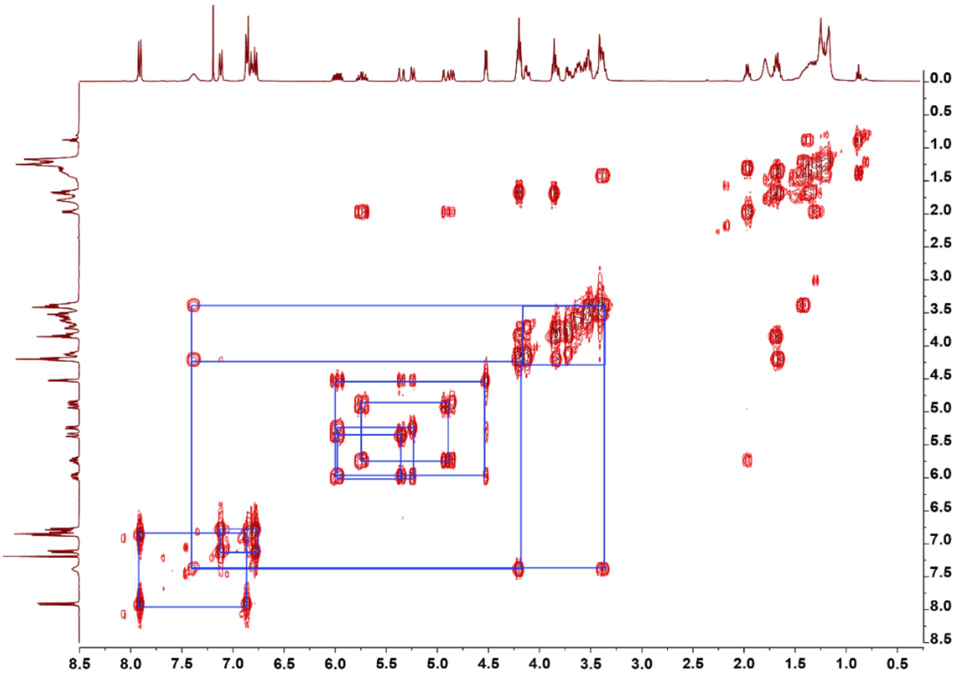

Supplementary Fig. 17 COSY NMR spectrum (400 MHz, $CDCl_3$, 298K) of [2]rotaxane **2**.

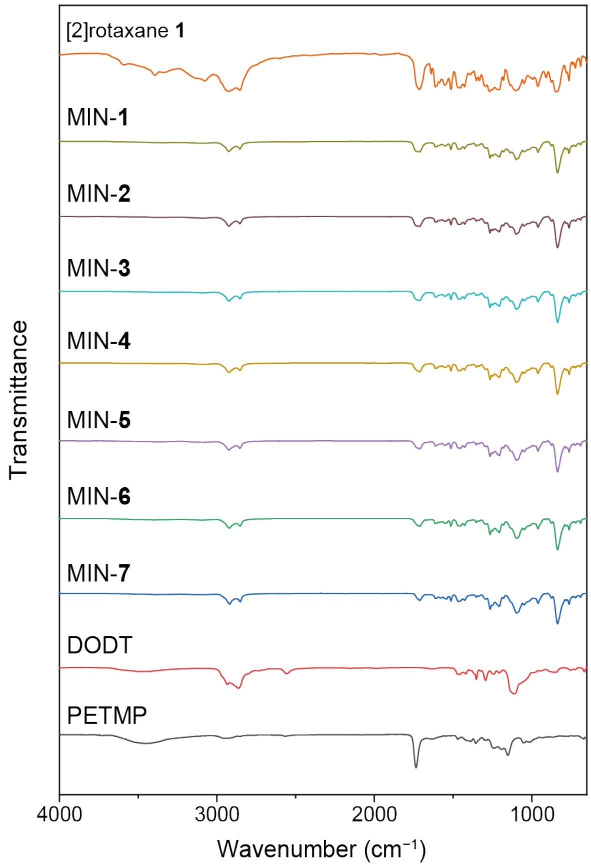

Supplementary Fig. 18 FTIR spectra of [2]rotaxane **1**, MINs-**1–7**, DODT and PETMP.

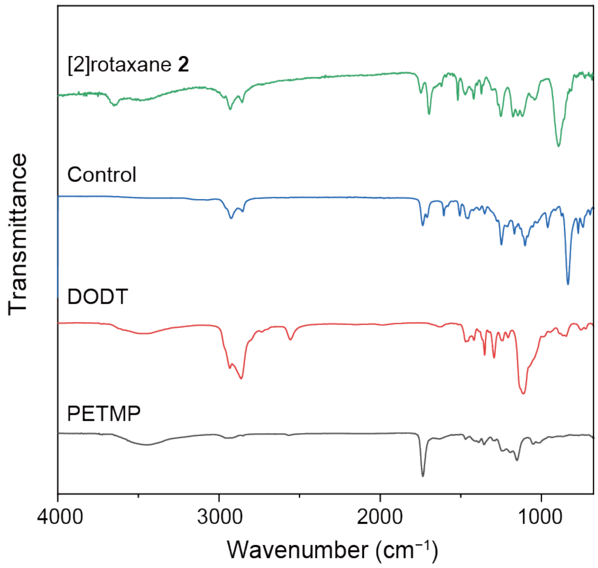

Supplementary Fig. 19 FTIR spectra of [2]rotaxane **2**, control, DODT and PETMP.

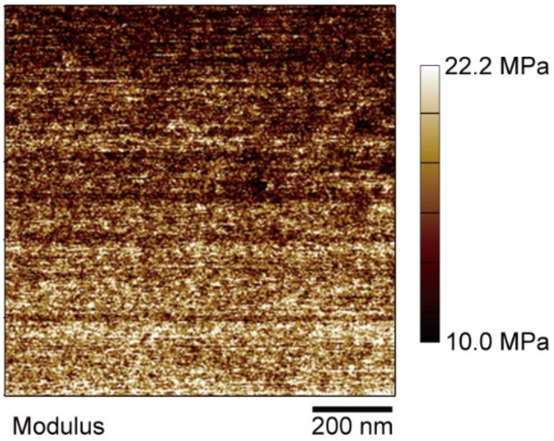

Supplementary Fig. 20 AFM modulus image of MIN-**4**.

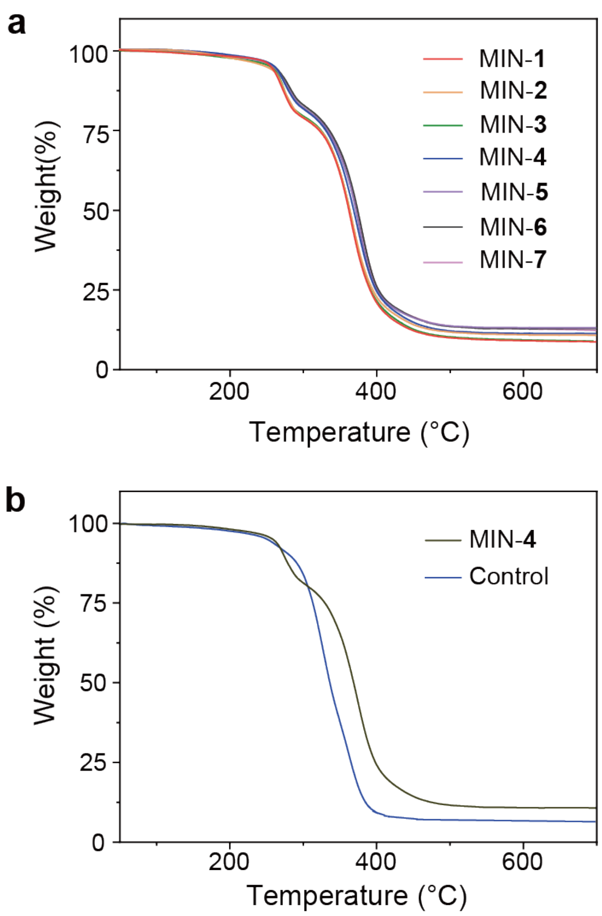

Supplementary Fig. 21 TGA curves of (a) MINs-**1**−**7** and (b) MIN-**4** and control recorded under N_2 flow (50 mL/min) with a heating rate of 10 °C/min.

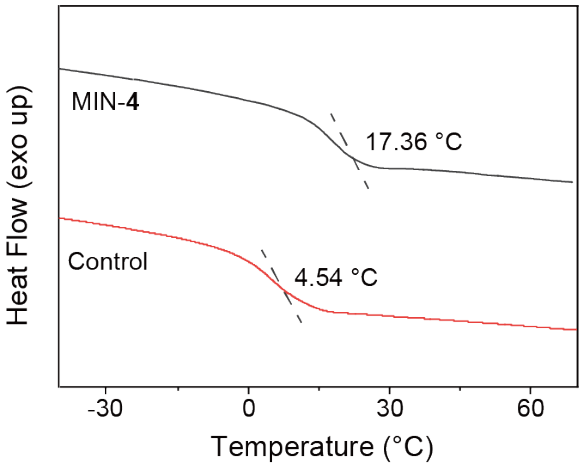

Supplementary Fig. 22 DSC curves of MIN-**4** and control measured by the second heating scan from –40 to 120 °C with a rate of 10 °C/min.

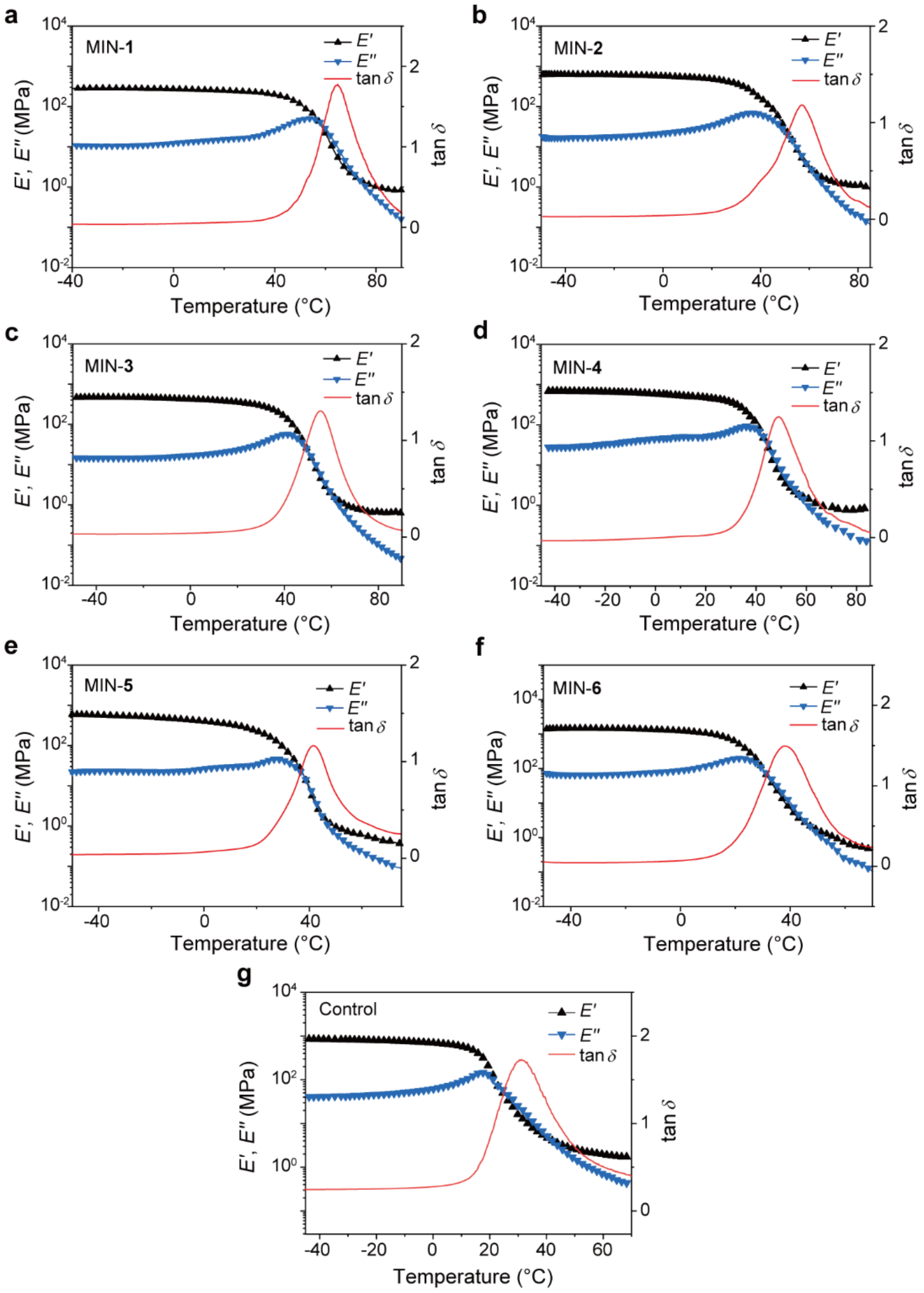

Supplementary Fig. 23 (a–g) DMA temperature ramps of MINs-**1**−**6** and control from −40 to 80 °C with a heating rate of 5.0 °C/min.

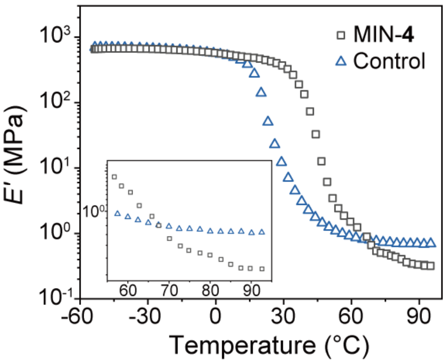

Supplementary Fig. 24 DMA temperature ramps of MIN-**4** and control from −50 to 100 °C with a heating rate of 5.0 °C/min.

Supplementary Table 2 The mechanical properties of MINs-**1**−**6** and control.

	Stress (MPa)	Strain (%)	Toughness (MJ/m^3)	Young's modulus (MPa)
MIN-**1**	29.5	31.6	7.00	281
MIN-**2**	16.5	119	15.1	189
MIN-**3**	10.6	287	24.7	120
MIN-**4**	14.3	542	47.2	101
MIN-**5**	14.5	520	40.0	66.4
MIN-**6**	6.27	337	11.1	37.2
control	1.26	268	1.70	1.68

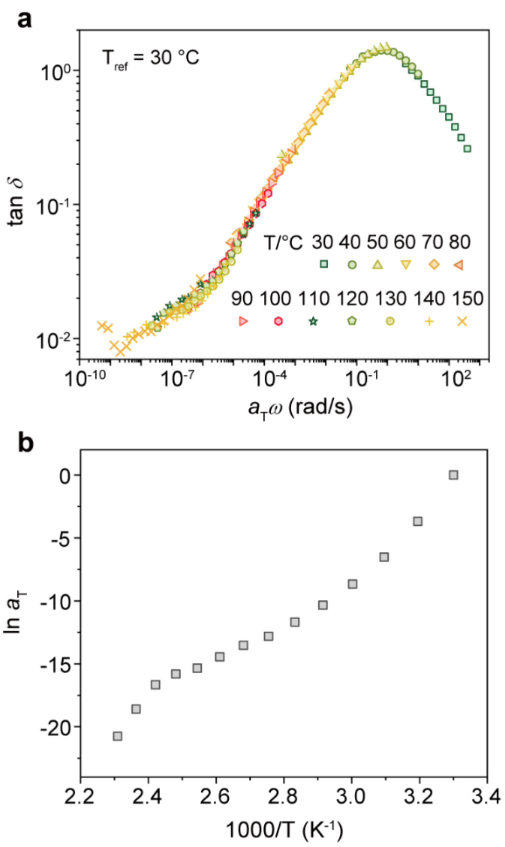

Supplementary Fig. 25 (a) Loss tangent (tan δ) versus frequency of MIN-**4**. (b) Temperature dependence of horizontal shift factors (a_T) of the master curves of MIN-**4**.

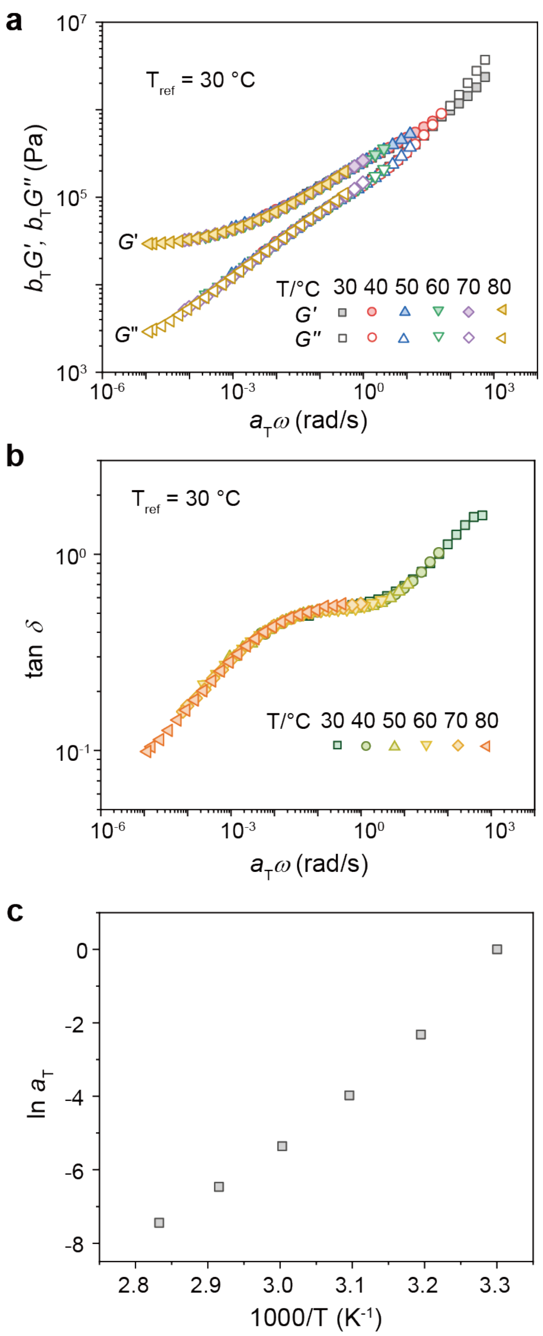

Supplementary Fig. 26 (a) Master curves of control at a reference temperature of 30 °C. (b) Loss tangent (tan δ) versus frequency of control. (c) Temperature dependence of horizontal shift factors (a_T) of the master curves of control.

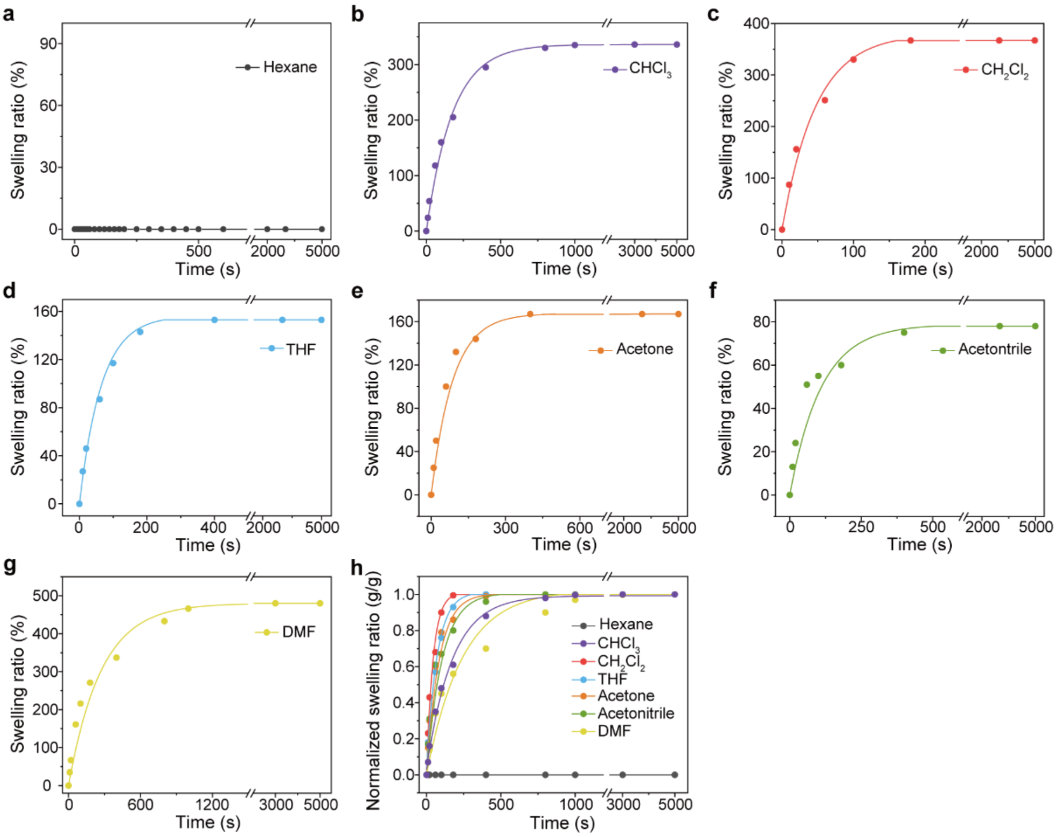

Supplementary Fig. 27 (a–g) Swelling ratios of MIN-**4** in seven different solvents, respectively. (h) Normalized swelling ratio curves of MIN-**4**.

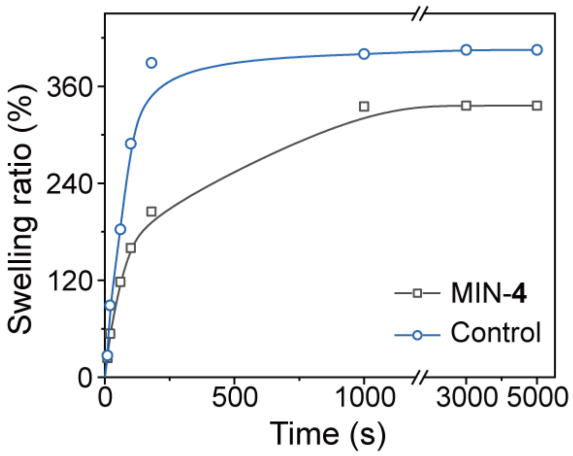

Supplementary Fig. 28 Swelling ratios of MIN-**4** and control in $CHCl_3$.

The swelling ratio of MIN-**4** and control in chloroform were 336% and 405%, respectively. Theoretically, the swelling ratios of MIN-**4** and control should be close because they have the same cross-linking density. As for their different swelling ratios, we infer that it is more likely due to their different glass transition temperatures (T_g). The T_g of control (4.50 ºC) was much lower than that of MIN-**4** (17.4 ºC). It meant that the movement of chain segments in control was much easier, which allowed the network to absorb more solvents, thus leading to a higher swelling ratio.

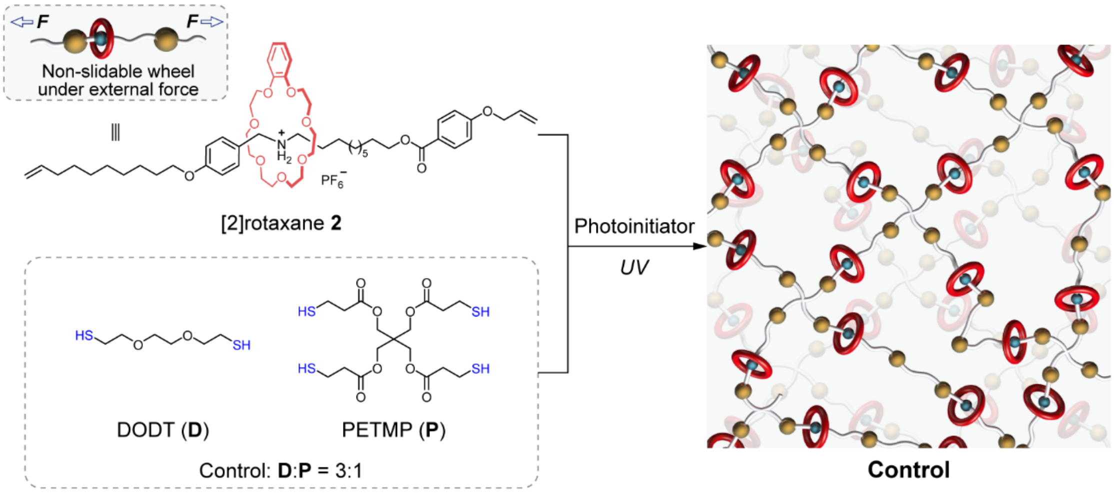

Supplementary Fig. 29 Chemical structures and schematic representation of the formation of control through thiol–ene click chemistry of the [2]rotaxane **2**, DODT and PETMP.

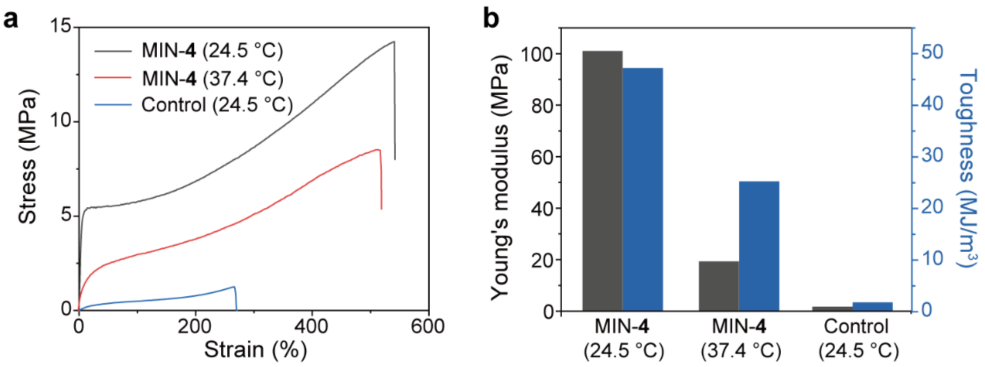

Supplementary Fig. 30 (a) Stress–strain curves of MIN-**4** and control at different temperatures with a deformation rate of 100 mm/min. (b) Young's moduli and toughness of MIN-**4** and control at different temperatures calculated from their corresponding stress–strain curves.

To exclude the influence of T_g on the mechanical properties of MINs, we conducted tensile tests for the two samples at the temperatures higher than their T_gs at the same degree. The test temperatures were set to 37.4 ºC for MIN-**4** (T_g = 17.4 ºC) and 24.5 ºC for the control (T_g = 4.50 ºC). In addition, the tensile test for MIN-**4** at 24.5 ºC was also performed. Firstly, the results for MIN-**4** at 24.5 ºC and 37.4 ºC showed that their Young's moduli were 101 and 19.3 MPa, and toughness were 47.2 and 25.2 MJ/m^3, respectively. These results verified the conclusion that the mechanical properties of MINs were largely affected by the test temperatures. In spite of this, the mechanical properties of the MIN-**4** measured at 37.4 ºC were much superior to those of the control at 24.5 ºC. The Young's modulus was 19.3 MPa for MIN-**4** (37.4 ºC) and 1.68 MPa for control (24.5 ºC), and toughness was 25.2 MJ/m^3 for MIN-**4** (37.4 ºC) and 1.70 MJ/m^3 for control (24.5 ºC). Moreover, the breaking strain were 517% and 268%, and the breaking stress were 8.48 and 1.26 MPa, for MIN-**4** (37.4 ºC) and control (24.5 ºC), respectively. Therefore, we can conclude that T_g played an important role in the mechanical properties of MINs as for general elastomers. Meanwhile, the role of integration and amplification effect on the mechanical properties of MINs was further verified by the comparison of the tensile tests for MIN-**4** (37.4 ºC) and control (24.5 ºC).

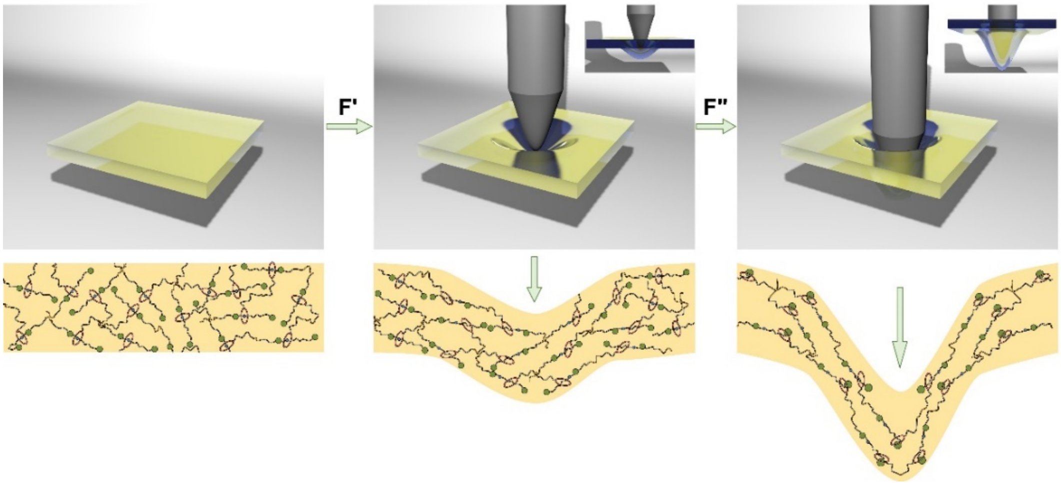

Supplementary Fig. 31 Cartoon representation of the puncture process of MIN-**4**.

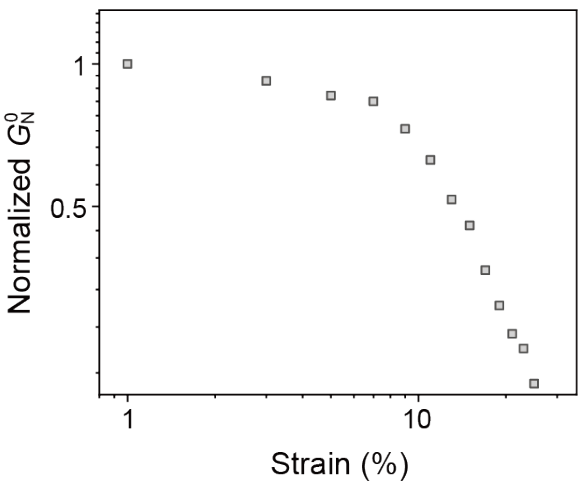

Supplementary Fig. 32 The partial normalized average plateau modulus (G_N^0) of MIN-**4** as a function of shear strain in 60−100 s based on the stress relaxation experiments.

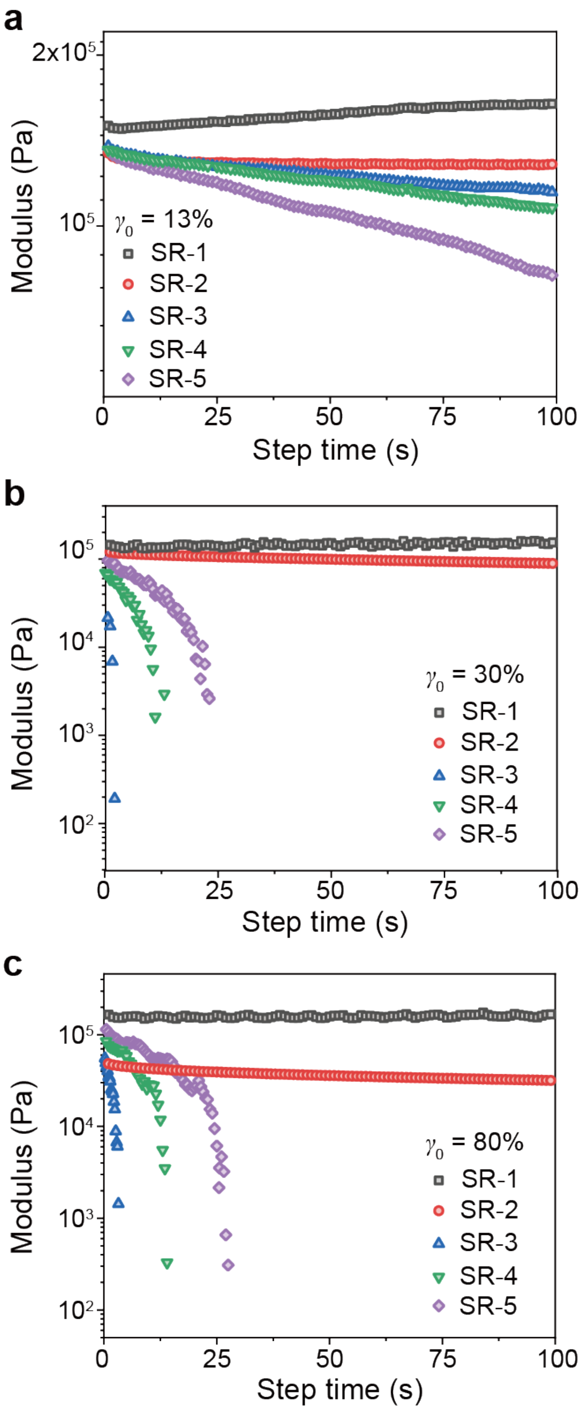

Supplementary Fig. 33 Consecutive stress relaxation from SR-1 to SR-5 of MIN-**4** with the defined strains of (a) 13%, (b) 30%, and (c) 80%, respectively.

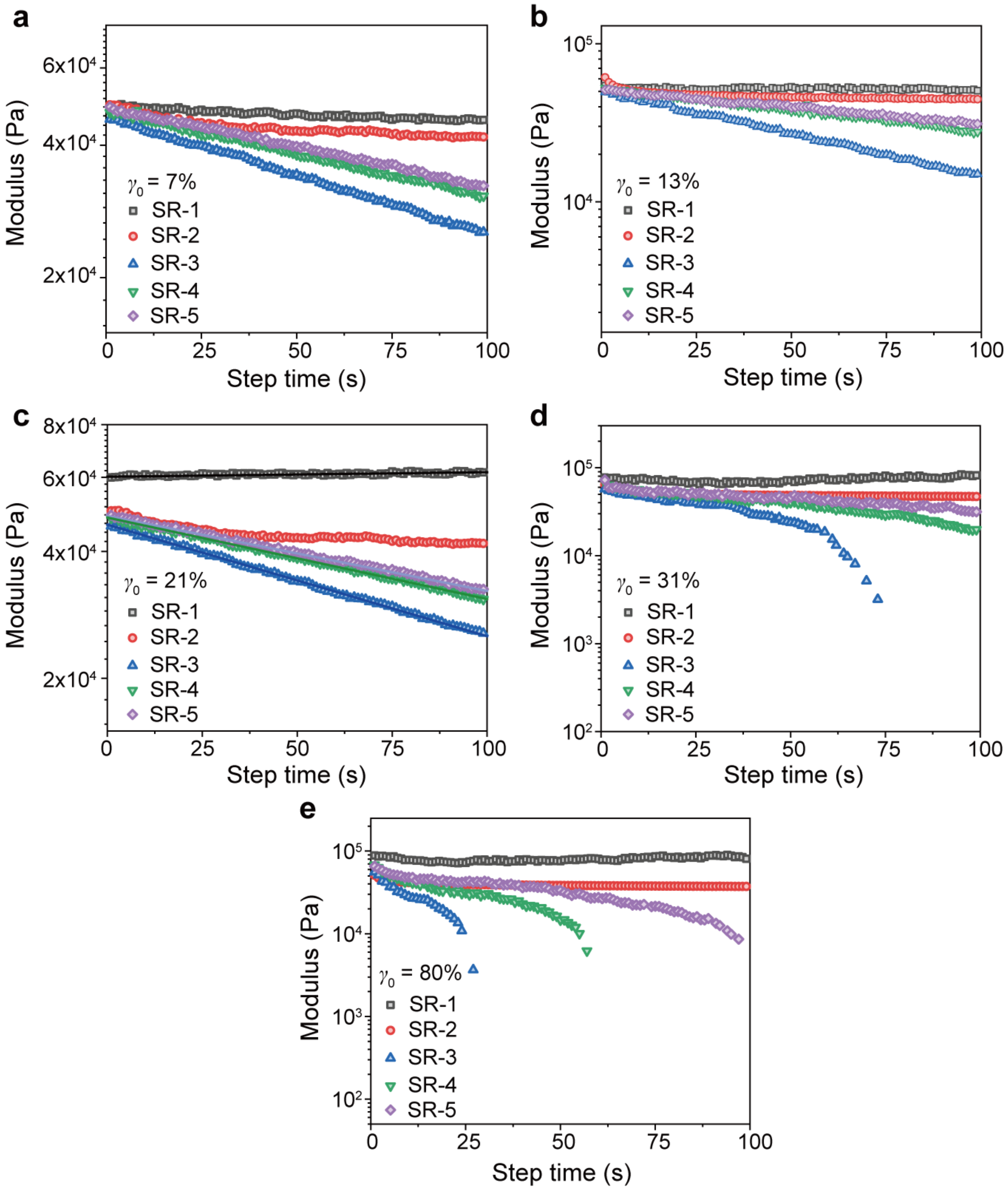

Supplementary Fig. 34 Consecutive stress relaxation from SR-1 to SR-5 of control with the defined strains of (a) 7%, (b) 13%, (c) 21%, (d) 31%, and (e) 80%, respectively.

0Å

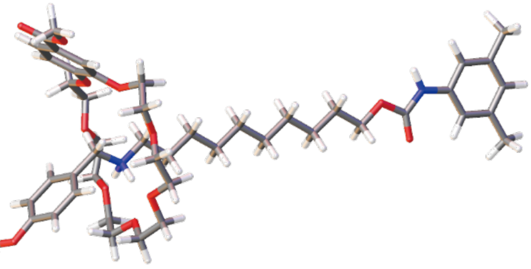

5Å

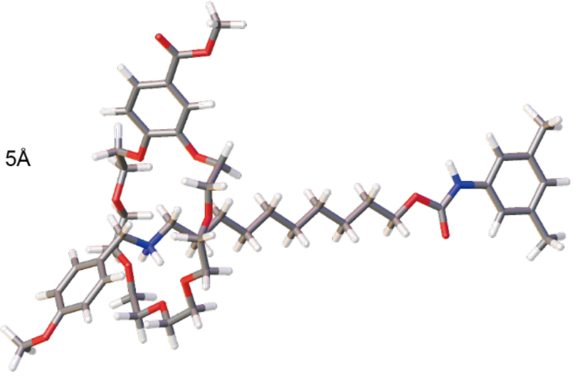

10Å

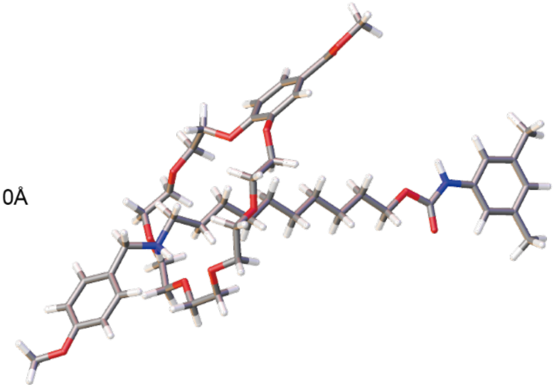

11Å

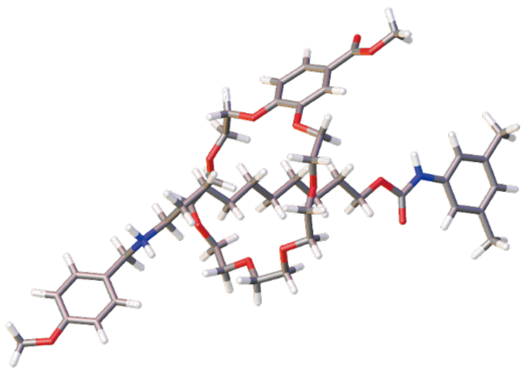

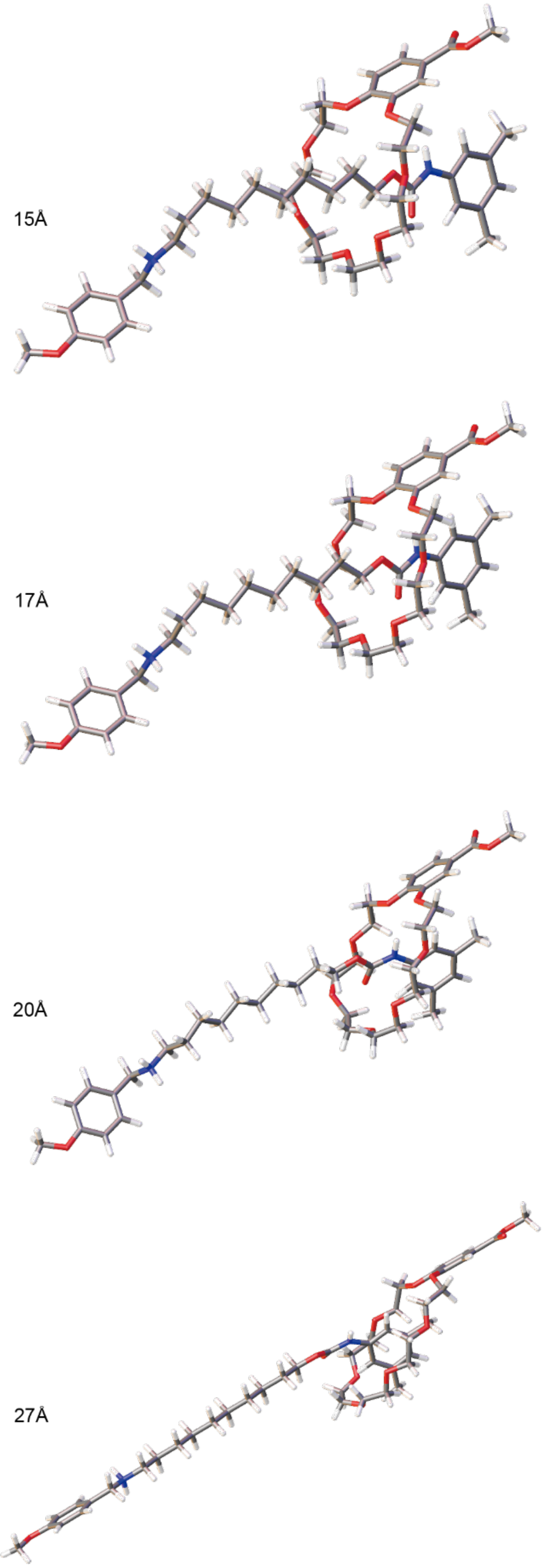

Supplementary Fig. 35 CoGEF structures of a segment of [2]rotaxane **1** with different displacements.

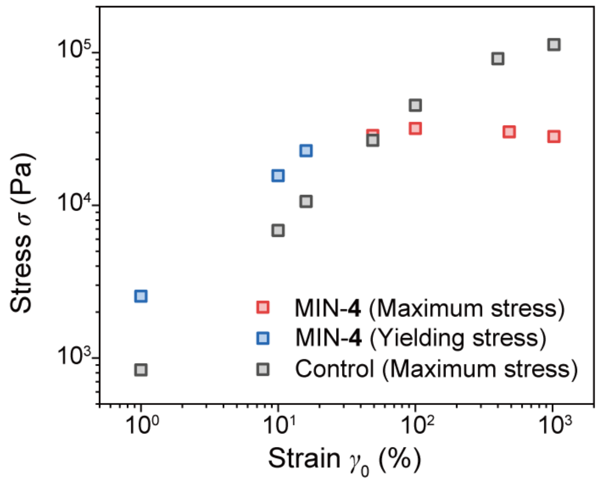

Supplementary Fig. 36 The shear stress (σ) versus shear strain (γ_0) based on the method described in Figure 6a.

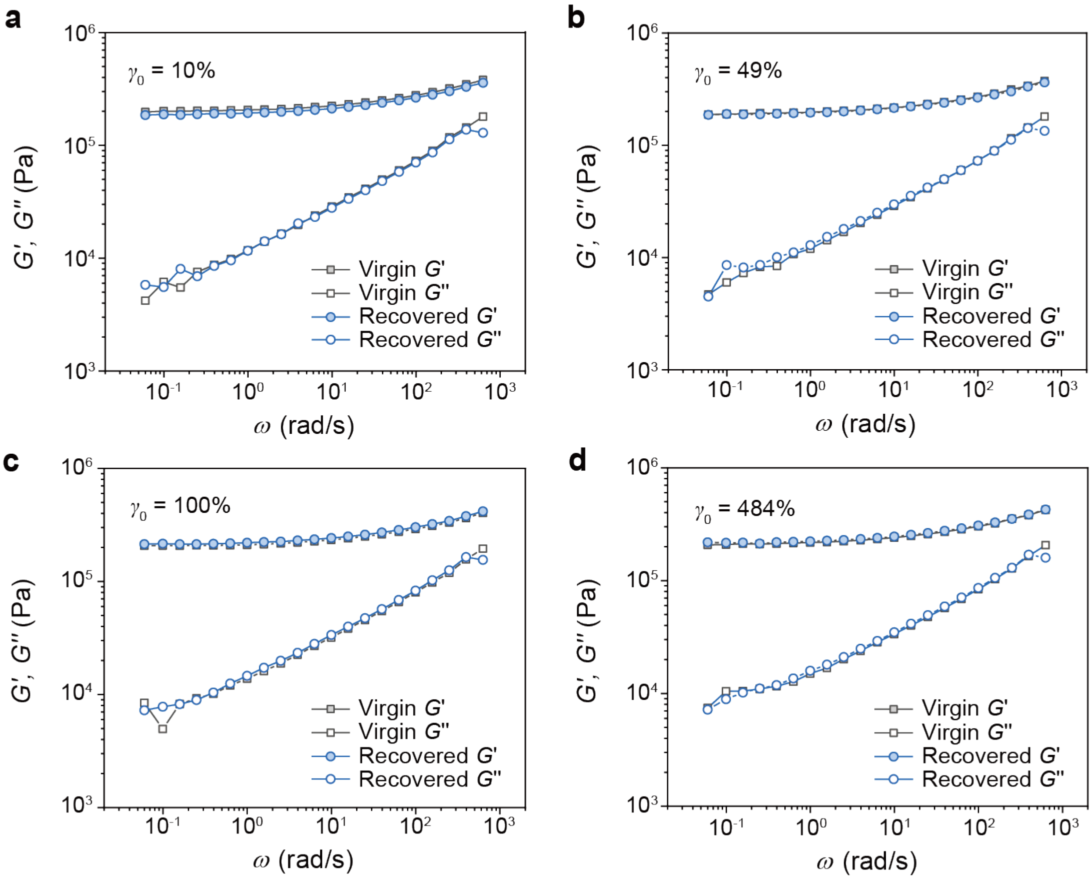

Supplementary Fig. 37 (a–d) The virgin and recovered *G′* and *G″* of MIN-**4** versus frequency (*ω*) at different shear strains obtained by the method described in Figure 6a.

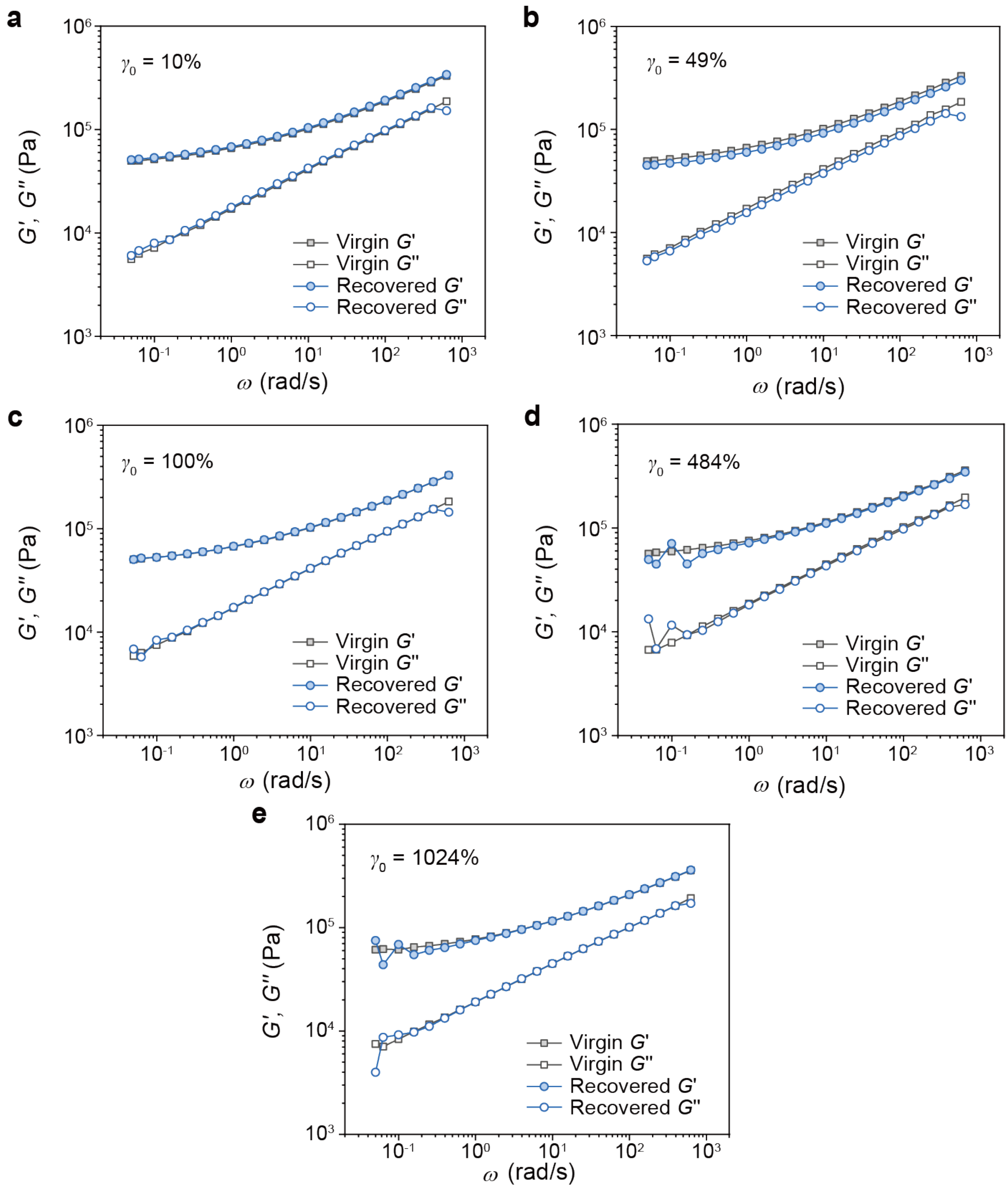

Supplementary Fig. 38 (a–e) The virgin and recovered G' and G'' of control versus frequency (ω) at different shear strains obtained by the method described in Figure 6a.

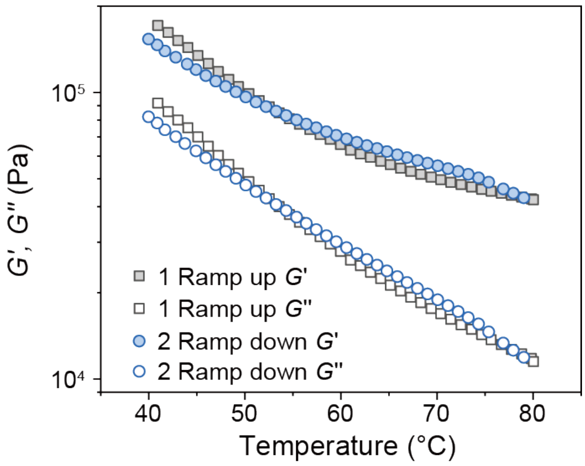

Supplementary Fig. 39 Cyclic temperature ramp curves of control ranging from 40 to 80 °C with a heating/cooling rate of 5 °C/min.

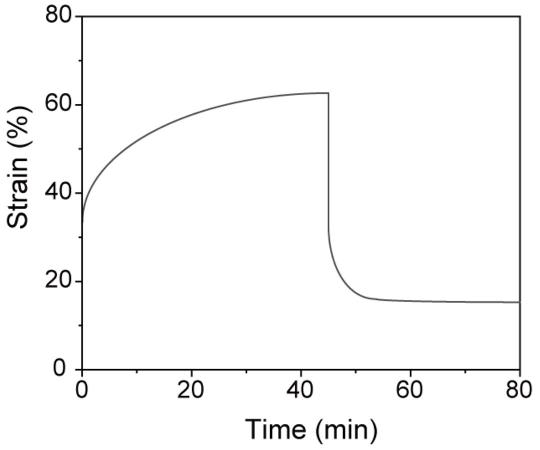

Supplementary Fig. 40 Creep and recovery curve of control at a constant stress of 0.2 MPa.

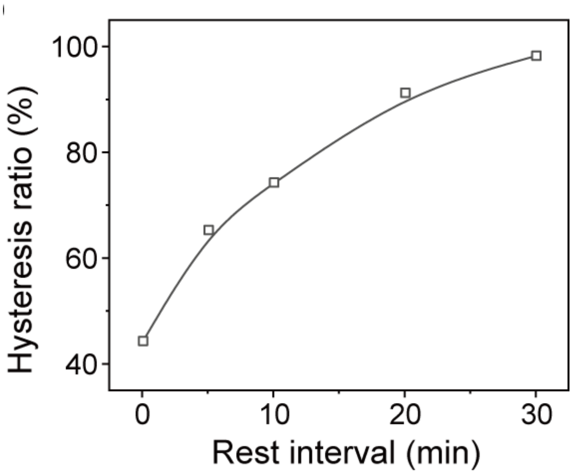

Supplementary Fig. 41 Hysteresis ratio of MIN-**4** for each cycle calculated from the results of the cyclic tensile tests in Fig. 6e.

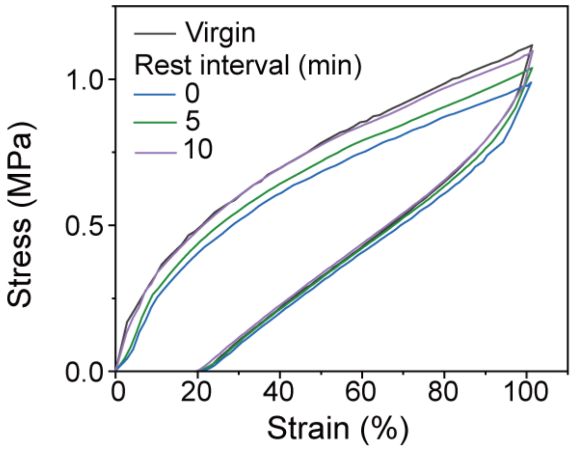

Supplementary Fig. 42 Cyclic tensile test curves of control loaded at a strain of 100% with rest intervals ranging from 0 to 10 min.

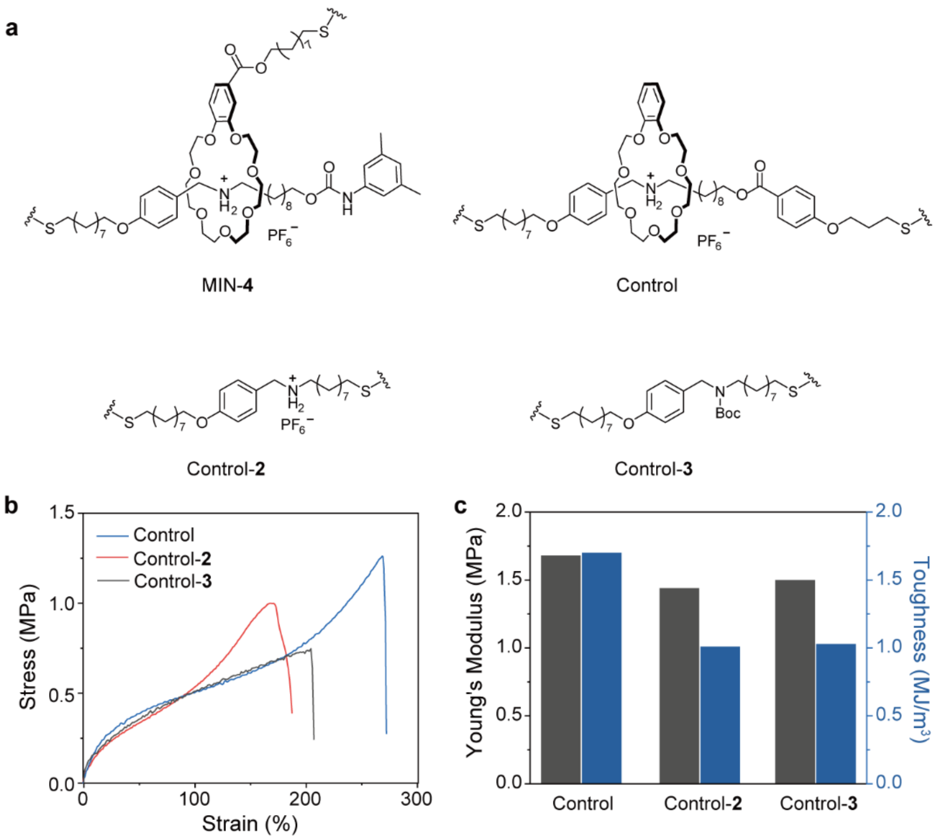

Supplementary Fig. 43 (a) Chemical structures of MIN-**4** and three controls. (b) Stress–strain curves of control, control-**2** and control-**3** recorded with a deformation rate of 100 mm/min. (c) Young's modulus and toughness of control, control-**2** and control-**3** calculated based on their stress–strain curves.

These experiments were designed to clarify a fact that whether the ionic interactions of ammonium salt play a role in the mechanical properties of MINs. We deduce that the ionic interactions between inter-chains could be discussed under the two situations: (1) when the B21C7 wheel binds to the secondary ammonium salt (MINs without deformation), the positive sites are less likely to interact with each other, because they are surrounded by the bulky crown ethers; (2) upon external force, the sliding of the B21C7 wheel along the axle could release the ammonium sites. In this case, the formation of ionic interactions between exposed ammonium groups might be possible. However, direct characterization of the transiently formed ionic interactions is very difficult. Corresponding study could be assisted by the comparison of reasonable controls.

For this purpose, two additional control samples were designed and synthesized (Fig. S41a). The control-**2** sample was a polymer whose repeating unit was only the axle bearing ammonium site rather than [2]rotaxane. Its naked ammonium sites can be used to evaluate the possibility of ionic interactions during stretching of MINs. The control-**3** sample was identical to that of control-**2** except that the ammonium was neutralized and protected by a Boc group. The stress–strain curves of the original control, control-**2** and control-**3** were exhibited in Fig. S41b, and corresponding data of Young's modulus and toughness were summarized in Fig. S41c. It is reasonable to deduce that if the ionic interactions are present in control-**2**, its Young's modulus should be higher than that of control and control-**3** because the interactions could endow the control-**2** with additional physical cross-linking. For the same reason, the toughness of control-**2** might be also superior to that of control and control-**3**, benefitting from the energy dissipation arising from dissociation of the physical cross-linking of ionic interactions. However, such phenomena were not observed in the measurements. Young's moduli for control, control-**2** and control-**3** were 1.68, 1.44 and 1.50 MPa, and toughness for them were 1.70, 1.01 and 1.03 MJ/m^3, respectively. Therefore, the present results don't support the formation of ionic interactions between the chains of MINs.

Supplementary References

1. Yan, X., Zhou, M., Chen, J., Chi, X., Dong, S., Zhang, M., Ding, X., Yu, Y., Shao, S., Huang, F. Supramolecular polymer nanofibers *via* electrospinning of a heteroditopic monomer. *Chem. Commun. 47*, 7086–7088 (**2011**).

2. Jiang, T., Wang, W., Wu, X., Wu, W., Bai, H., Ma, Z., Shen, Y., Yang, K., Li, M. Discovery of new substrates for *Lux*AB bacterial bioluminescence. *Chem Biol Drug Des*. *88*, 197–208 (**2016**).

3. Nicolaou, K. C., Cho, S. Y., Hughes, R., Nicolas, W., Smethurst, C., Labischinski, H. Solid- and solution-phase synthesis of vancomycin and vancomycin analogues with activity against vancomycin-resistant bacteria. *Chem. Eur. J. 7*, 3798–3823 (**2001**).

4. Harnoy, A. J., Slor, G., Tiroshb, E., Amir, R. J. The effect of photoisomerization on the enzymatic hydrolysis of polymeric micelles bearing photo-responsive azobenzene groups at their cores. *Org. Biomol. Chem. 14*, 5813–5819 (**2016**).

5. Beyer, M. K. The mechanical strength of a covalent bond calculated by density functional theory. *J. Chem. Phys. 112*, 7307−7312 (**2000**).

6. Gaussian 09, Revision B.01, M. J., Frisch, G. W., Trucks, H. B., Schlegel, G. E., Scuseria, M. A., Robb, J. R., Cheeseman, G., Scalmani, V., Barone, B., Mennucci, G. A., Petersson, H., Nakatsuji, M., Caricato, X., Li, H. P., Hratchian, A. F., Izmaylov, J., Bloino, G., Zheng, J. L., Sonnenberg, M., Hada, M., Ehara, K., Toyota, R., Fukuda, J., Hasegawa, M., Ishida, T., Nakajima, Y., Honda, O., Kitao, H., Nakai, T., Vreven, J. A., Montgomery, Jr. J. E., Peralta, F., Ogliaro, M., Bearpark, J. J., Heyd, E., Brothers, K. N., Kudin, V. N., Staroverov, T., Keith, R., Kobayashi, J., Normand, K., Raghavachari, A., Rendell, J. C., Burant, S. S., Iyengar, J., Tomasi, M., Cossi, N., Rega, J. M., Millam, M., Klene, J. E., Knox, J. B., Cross, V., Bakken, C., Adamo, J., Jaramillo, R., Gomperts, R. E., Stratmann, O., Yazyev, A. J., Austin, R., Cammi, C., Pomelli, J. W., Ochterski, R. L., Martin, K., Morokuma, V. G., Zakrzewski, G. A., Voth, P., Salvador, J. J., Dannenberg, S., Dapprich, A. D., Daniels, O., Farkas, J. B., Foresman, J. V., Ortiz, J., Cioslowski, D. J., Fox, Gaussian, Inc. Wallingford CT, (**2010)**.

7. Becke, A. D. Density-functional thermochemistry. III. The role of exact exchange. *J. Chem. Phys*. *98*, 5648−5652 (**1993**).

8. Lee, C., Yang, W., Parr, R. G. Development of the Colle-Salvetti correlation-energy formula into a functional of the electron density. *Phys. Rev. B. 37*, 785−789 (**1988**).

9. Petersson, G., Al-Laham, M. A. A complete basis set model chemistry. II. Open-shell systems and the total energies of the first-row atoms. *J. Chem. Phys*. *94*, 6081−6090 (**1991**).
